# Niche-specification of aerobic 2,4-dichlorophenoxyacetic acid biodegradation by *tfd*-carrying bacteria in the rice paddy ecosystem

**DOI:** 10.3389/fmicb.2024.1425193

**Published:** 2024-08-23

**Authors:** Tran Quoc Tuan, Panji Cahya Mawarda, Norhan Ali, Arne Curias, Thi Phi Oanh Nguyen, Nguyen Dac Khoa, Dirk Springael

**Affiliations:** ^1^Division of Soil and Water Management, Department of Earth and Environmental Sciences, KU Leuven, Leuven, Belgium; ^2^Department of Molecular Biology, Institute of Food and Biotechnology, Can Tho University, Can Tho, Vietnam; ^3^Research Center for Applied Microbiology, National Research and Innovation Agency Republic of Indonesia (BRIN), KST Samaun Sadikun, Bogor, Indonesia; ^4^Department of Biology, College of Natural Sciences, Can Tho University, Can Tho, Vietnam

**Keywords:** rice paddy, 2,4-D biodegradation, niche-specification, aerobic compartments, molecular markers, *TfdA* gene

## Abstract

This study aimed for a better understanding of the niche specification of bacteria carrying the *tfd*-genes for aerobic 2,4-dichlorphenoxyacetic acid (2,4-D) degradation in the rice paddy ecosystem. To achieve this, a dedicated microcosm experiment was set up to mimic the rice paddy system, with and without 2,4-D addition, allowing spatial sampling of the different rice paddy compartments and niches, i.e., the main anaerobic bulk soil and the aerobic surface water, surface soil, root surface and rhizosphere compartments. No effect of 2,4-D on the growth and morphology of the rice plant was noted. 2,4-D removal was faster in the upper soil layers compared to the deeper layers and was more rapid after the second 2,4-D addition compared to the first. Moreover, higher relative abundances of the 2,4-D catabolic gene *tfdA* and of the mobile genetic elements IncP-1 and IS*1071* reported to carry the *tfd*-genes, were observed in surface water and surface soil when 2,4-D was added. *tfdA* was also detected in the root surface and rhizosphere compartment but without response to 2,4-D addition. While analysis of the bacterial community composition using high-throughput 16S rRNA gene amplicon sequencing did not reveal expected *tfd*-carrying taxa, subtle community changes linked with 2,4-D treatment and the presence of the plant were observed. These findings suggest (i) that the surface soil and surface water are the primary and most favorable compartements/niches for *tfd*-mediated aerobic 2,4-D biodegradation and (ii) that the community structure in the 2,4-D treated rice paddy ecosystem is determined by a niche-dependent complex interplay between the effects of the plant and of 2,4-D.

## 1 Introduction

Rice (*Oryza sativa* L.) is one of the most important food crops, feeding over half of the world’s population. Nearly 95% of the world’s rice is produced from paddy rice farming (Muthayya et al., 2014). Intensive monoculture in rice paddy farming makes rice plants highly vulnerable to pests and diseases, leading to an increasing reliance on pesticides to sustain high production yields. However, only 10% of the applied pesticide load reaches its target, while the remaining is released into the environment via volatilization, run-off, drainage, and leaching (Mogul et al., 1996). The released pesticides can directly affect non-target organisms and contaminate water resources, resulting in human and overall ecosystem health issues and threatening economic activities such as integrated rice-fish farming and aquaculture (Berg, 2002; Comoretto et al., 2008; Anyusheva et al., 2012; Ortiz-Hernández et al., 2013; Dang et al., 2017). Therefore, understanding the fate of pesticides and the underlying mechanisms in rice paddy ecosystems, particularly the removal processes, is of major importance for ecological risk assessment, health security, and economic reasons.

Microbial biodegradation is considered the main route that determines the environmental fate of pesticides in soils (Schwarz et al., 2022). Processes and microbiota involved in the biodegradation of various pesticides have been reported especially in upland crops, but less is known about the processes that occur during paddy rice cultivation (Ortiz-Hernández et al., 2013; Bose et al., 2021; Raffa and Chiampo, 2021). Microbial degradation of pesticides primarily occurs under aerobic conditions. Rice cultivation in paddy fields involves flooded conditions which are established before rice planting and maintained during the period of plant growth. Once flooded, the oxygen in the bulk soil is consumed rapidly due to the digestion of organic residues from the rice plant of the previous cultivation period, and the soil is transformed into a dominantly anaerobic system (Liesack et al., 2000; Tomco et al., 2010). However, several aerobic compartments that provide potential niches for aerobic pesticide biodegradation can be recognized. These include the floodwater and the surface layer of the soil where oxygen is delivered by diffusion from the air and/or by the photosynthetic activity of algae and aquatic plants (Roger et al., 1993). In addition, oxygen is released at the level of the root surface and rhizosphere through leakage from aerenchymatous tissue and at sufficient rates to support aerobic microbial processes like aerobic decomposition of organic matter, nitrification, and methane oxidation (Roger et al., 1993; Van Bodegom et al., 2001). Aerobic and anaerobic pesticide biodegradation has been reported to occur simultaneously under flooded conditions in rice paddies (Tomco et al., 2010; Vasquez et al., 2011; Mulligan et al., 2016) but it is unknown which niches are particularly involved and how the application of pesticides affects the microbial communities in the different niches.

2,4-dichlorophenoxyacetic acid (2,4-D) is a herbicide whose chemical structure and biological activity resemble that of the plant hormone auxin. The compound induces abnormal growth causing plant death when supplied at high concentrations and is commonly used as a selective herbicide to control broadleaf weeds (Song, 2014). In rice paddy fields, 2,4-D is used at the post-emergence stage of the weeds, 15–20 days after sowing the rice plant seeds, or at 7–10 days after transplanting rice seedlings (International Rice Research Institute, 2007). While 2,4-D biodegradation has been shown to occur under anaerobic conditions (Zipper et al., 1999; Wu et al., 2009; Ha, 2018), the most well-known bacterial route of 2,4-D biodegradation is the aerobic pathway encoded by the *tfd* genes. This pathway has been studied extensively and especially characterized in *Cupriavidus necator* JMP134 (Kumar et al., 2016). The *tfdA* gene, encoding an α-ketoglutarate-dependent dioxygenase, initiates the pathway by converting 2,4-D into 2,4-dichlorophenol (Fukumori and Hausinger, 1993a,b). The latter is further metabolized to Krebs cycle intermediates by enzymes encoded by the *tfdBCDEF* gene cluster (Laemmli et al., 2000; Perez-Pantoja et al., 2000, 2003; Plumeier et al., 2002; Kumar et al., 2016). The *tfd* genes are often located on mobile gene elements such as on IncP-1 plasmids in association with the insertion sequence IS*1071*. Likely, these mobile elements contributed to the adaptation process of the involved organisms and community toward 2,4-D catabolism by horizontal gene transfer (HGT) (Kim et al., 2013; Nguyen et al., 2019). The *tfdA* gene is further used as a genetic marker for interrogating the abundance and activity of 2,4-D catabolic bacteria in environmental samples by quantitative PCR and reverse transcriptase-PCR (Baelum and Jacobsen, 2009; Han et al., 2014; Mierzejewska et al., 2020). Based on its sequence, *tfdA* is phylogenetically classified into three distinct classes, i.e., classes I, II, and III (Baelum et al., 2010). Class I and class III are present in various bacterial taxa, whereas class II is primarily found in *Burkholderia* spp. Class I and class III *tfdA* genes are more often located on self-transmissible broad host spectrum plasmids compared to class II *tfdA* resulting in being more readily distributed by HGT (Top et al., 1995; DiGiovanni et al., 1996; McGowan et al., 1998; de Lipthay et al., 2001). Studies investigating the ecology of bacterial 2,4-D biodegradation, mainly in upland soils, showed a correlation between the *tfdA* gene relative abundances and 2,4-D mineralization capacity in the studied soils and the pathway likely composes a primary route of 2,4-D biodegradation in 2,4-D treated agricultural systems (Baelum and Jacobsen, 2009; Baelum et al., 2012). Recently, several 2,4-D catabolic bacterial strains were isolated from rice paddy ecosystems in Vietnam and Nigeria. They all are members of closely related β-proteobacterial genera like *Burkholderia* sp., *Cupriavidus* sp., and *Ralstonia* sp. Moreover, they all carried the *tfd* operon for 2,4-D catabolism including the class I *tfdA* gene suggesting that aerobic biodegradation mediated by the *tfd* operon contributes to 2,4-D biodegradation in rice paddy ecosystems, despite the mainly anaerobic character of the bulk soil (Nguyen et al., 2019; Muhammad et al., 2023). However, it can be hypothesized that *tfd*-carrying bacteria establish and inhabit one or more of the above mentioned aerobic compartments and niches.

This study aims to acquire a better understanding of the microbial ecology of *tfd*-carrying bacteria in the rice paddy ecosystem and particularly to identify the niches that sustain the *tfd*-governed process of 2,4-D biodegradation. To this end, rice microcosms were set up that mimic the rice paddy ecosystem and allow the spatial separation of the oxic and anoxic compartments. The microcosms contained soil collected from rice fields in the Mekong delta of Vietnam, the largest rice producing area of the country. The sampled rice paddy fields had a history of 2,4-D application. Five environmental compartments of the rice paddy system representing different niches of potential pesticide biodegradation were considered, i.e., surface water, surface soil (upper sediment layer), rhizosphere, root surface, and bulk soil. The microcosms were treated or non-treated with 2,4-D and niche-bound relative abundances of the *tfdA* gene and genes associated with HGT of the *tfd* genes, i.e., IncP-1 plasmids and IS*1071*, were determined. Moreover, the bacterial community composition was determined using high-throughput 16S rRNA gene amplicon sequencing. We hypothesized that the relative abundances of the *tfdA* gene and its associated MGEs would be particularly high in the aerobic niches and increase when 2,4-D was supplied. Moreover, we expected that the bacterial community composition would differ between the different niches with taxa previously associated with *tfd*-mediated 2,4-D biodegradation, like *Burkhholderia-*related taxa, responding to 2,4-D addition.

## 2 Materials and methods

### 2.1 Paddy rice microcosm setup and sample collection

Triplicate microcosms per condition were set up in stainless steel cylinders (6 cm diameter × 16 cm length) equipped with a screw cap at the bottom and nine water sampling ports along the length of the cylinder at 1.25 cm vertical distance from each other (see Supplementary Figure S1). The sampling ports were equipped with syringes and rhizon samplers (porous part: 2.5 cm, outer diameter: 2.5 mm, pore size of membrane: 0.12–0.18 μm) (Rhizosphere Research Products) for water sampling. Implemented conditions were with and without rice plant and with and without 2,4-D application, i.e., (A) “Soil + rice plant +2,4-D,” (B) “Soil + rice plant,” (C) “Soil +2,4-D,” and (D) “Soil.”

The soil used in the microcosms was collected from two drained rice paddy fields adjacent to each other and separated by a dike in My Xuyen ward in Soc Trang province in the Mekong delta of Vietnam (9^o^30′19.8″ N 105^o^54′08.3″ E). Both fields have a history of 2,4-D application. Five top soil samples (0–20 cm depth) were taken, i.e., four at the corners and one in the middle of each of the two rice fields on February 12, 2021, directly after the harvest of the rice plants. The fields were drained a few weeks before harvest but the soils were still moist when sampled. The soil samples from each field were stored as a mixture in a plastic bag and brought immediately to the laboratory. Rice straw and root residues were removed manually and the soils were grated using a cheese grater. Both soils were sieved (4 mm), mixed with each other and the mixture homogenized and stored at 4°C.

A root bag (5 × 18 cm), self-made from a nylon filter sheet (LytheE, China; mesh size: 25 μm), heat-sealed with an impulse sealer (American International Electric Inc.), and filled with 100 g of sieved (sterilized) soil (moisture content of 32%), was inserted in the middle of each cylinder after which the residual site volumes of the cylinder were filled with 230 g sieved soil. Afterward, the soil in the cylinder was saturated with sterile Milli-Q water 24 h before planting pre-germinated rice seeds inside the root bag of the microcosms intended to contain rice plants (one per microcosm). Pre-germinated paddy rice (*Oryza sativa* var. Jasmine 85) seeds were obtained by disinfection for 30 min in sterile hot Milli-Q water (54°C), soaking them for 24 h in sterile Milli-Q water to break rice dormancy and incubating them for 3 days at 25°C (Khoa et al., 2016). The microcosms were incubated in a plant growth chamber (Weiss Technik, Belgium) with day (12 h)/night (12 h), temperature setting of 25/20°C, relative humidity of 80%, and light intensity of 550–600 μmol.cm^−2^.s^−1^ and watered every day to maintain the surface water level at a height of 1.5 cm above the upper soil layer. Nutrients [(40.0 mg N – 13.3 mg P_2_O_5_–10.0 mg K_2_O)]/kg soil were added at four-time points. P_2_O_5_ was added 1 day before planting the pre-germinated rice seeds while N and K_2_O were added at 10, 20, and 40 days after planting (Khoa et al., 2016).

In 2,4-D treated microcosms, 12.5 mL of a 2,4-D solution (0.2 mg.mL^−1^) was added twice in each microcosm (similar to the rate recommended for application in the field), i.e., the first time at day 30 after planting the rice seed and the second time when 2,4-D supplied at the first time could not be detected anymore (day 57). At both time points, the 2,4-D solution was administered and distributed equally over the width and length of the cylinder by inserting a 20-mL syringe (Henke Sass Wolf, Germany) with a 120-mm needle (Braun, Germany) vertically into the soil column till a depth of around 12 cm and carefully moving it upwards while injecting 0.5 mL of the 2,4-D solution every 3 cm with the last injection in the surface water compartment. Injection was done at five surficial positions, i.e., four positions nearby the edge of the cylinder and one in the middle of the root bag. 2,4-D biodegradation was monitored by determining 2,4-D concentrations every day in water samples extracted from three sampling ports, i.e., at 3 cm (sampling port 1), 8 cm (sampling port 5), and 13 cm (sampling port 9). After centrifugating the samples at 15,000 rpm, 2,4-D concentrations were determined in the supernatant using UPLC on a Nexera (Shimadzu) apparatus equipped with a YMC Triart C18 column (inside diameter: 3 mm, length: 150 mm, particle size: 3 μm, pore size: 12 nm) (Achrom, Belgium) and a UV–VIS detector. The mobile phase was acetonitrile:H_2_O containing 2 mM NH_4_COOH (55:45) supplied at a flow rate of 0,4 mL.min^−1^. 2,4-D was detected at 210 nm at a retention time of 6.0 min (LOD = 0.2 mg.L^−1^ and LOQ = 0.5 mg.L^−1^). Moreover, the rice plants in plant-containing treatments were visually inspected for physiological differences and differences in growth behavior between microcosms with and without 2,4-D addition.

The different compartments of the microcosms were sampled when the concentration of 2,4-D added at the second application became undetectable. First, the water above the soil, representing the surface water layer niche, was collected using a 5-mL pipette without sucking any soil from the sediment layer, and filtered through a 0.2 μm filter membrane to collect the microbial biomass. Then, the screw cap at the bottom of the microcosm was removed and the complete soil column together with the rice plant was pushed out of the cylinder from its bottom by means of a dedicated ethanol (70%) sterilized piston. Half a centimeter of the top soil outside of the root bag representing the surface soil niche as well as the remaining soil representing the bulk soil niche, were collected. The rhizosphere and root surface niches were sampled as described previously (Edwards et al., 2015; Luo et al., 2017) with slight modifications. Briefly, the roots were removed from the root bag, and excess soil was manually removed, leaving approximately 1 mm of soil attached to the roots. The remaining soil was removed from the root sample by washing the roots in 100 mL phosphate-buffered saline (PBS) in a 250-mL flask on a horizontal shaker at 180 rpm for 10 min. The washed root was transferred to a new sterile 250-mL flask while the soil suspension was centrifuged for 10 min at 5,000 × g. The supernatant was discarded and the soil pellet, representing the rhizosphere niche, was retained. The washed root sample was sonicated at 50–60 Hz (Bransonic) in 100 mL PBS for 5 min to remove tightly adhering microbiota and the suspension was centrifuged for 10 min at 5,000 × g. The supernatant was discarded and the remaining pellet, representing the root surface niche, was collected. All niche samples, each of them well-mixed, were stored at −80°C prior to DNA extraction.

### 2.2 DNA extraction, end-point PCR, and quantitative PCR

Total DNA from all samples was extracted using the DNeasy PowerSoil Kit (Qiagen) following the manufacturer’s protocol. End-point PCR with specific primers (see Supplementary Table S1) was performed to assess the presence of class I, class II, and class III *tfdA* genes as described (Baelum and Jacobsen, 2009). The 25-μL reaction mixture consisted of Green buffer 1X (Qiagen), 0.1 mg.L^−1^ BSA, 200 μM of each dNTP, 0.625 U Taq polymerase, 0.1 μM of each primer, and 2 μL DNA template. Electrophoresis of the PCR amplicons was performed in a 1.5% agarose gel in TAE 1X and the fragments were visualized using gel red. Quantification of the bacterial 16S rRNA gene and the *tnpA* gene (as a marker for IS*1071*) was performed by SYBR Green real-time qPCR using the Absolute qPCR SYBR Green Mix (Thermoscientific). Quantification of class I *tfdA* and *korB* was performed by TaqMan real-time PCR using the EXPRESS qPCR SuperMix Universal (Invitrogen). The sequences of the used primers and Taqman probes as well as the temperature profiles for real-time qPCR, are shown in Supplementary Table S1. All qPCR reactions were done in a Rotor-Gene centrifugal real-time cycler (Corbett Research, Australia). Relative abundances of class I *tfdA*, *tnpA*, and *korB* were calculated as log *tfdA*/10^6^ 16S rRNA ratio.

### 2.3 16S rRNA gene amplicon sequencing and bacterial community analysis

The V3-V4 region of 16S rRNA gene was amplified from the DNAs extracted from the different niches using primer set 338F (5′-ACTCCTACGGGAGGCAGCAG-3′) and 806R (5′-GGACTACHVGGGTWTCTAAT-3′) (Caporaso et al., 2011) and the amplicons were sequenced using DNA Nanoball Sequencing by BGI Genomics, China. The Quantitative Insights Into Microbial Ecology (QIIME 2)^1^ software was used to process and analyze 16S rRNA gene sequences as described by Mawarda et al. (2022) (see Supplementary methods). The generated Amplicon Sequence Variant (ASV) table, rooted phylogenetic tree, and the taxonomy table were merged into a phyloseq object using phyloseq package v.1.36 (McMurdie and Holmes, 2013) in R v.3.6.0 (R Core Team, 2023) and ASVs identified as archaea, chloroplast, mitochondria, and uncharacterized taxa were removed from the phyloseq object. The data were rarefied to a sequencing depth of 28,461 and the remaining reads were used to examine the bacterial community composition among niches and treatments. α-diversity metrics (i.e., Species richness, Shannon diversity, Faith’s phylogenetic diversity) were calculated using phyloseq (McMurdie and Holmes, 2013) and Picante v.1.8.1 packages (Kembel et al., 2010). Bacterial community structure and composition variations (β-diversity) were evaluated by calculating Bray Curtis distance and visualized through Principle Coordinates Analysis (PCoA). Similarity percentages analysis (SIMPER) was performed to identify bacterial taxa that are responsible for these variations (Clarke, 1993).

### 2.4 Statistical analyses

The normality and the homogeneity of variance of the data were examined using Shapiro–Wilk’s test (Shapiro and Wilk, 1965) and Bartlett’s test (Bartlett and Fowler, 1937) to classify whether they fell into parametric and non-parametric categories. To assess significant differences (*p* < 0.05) in 2,4-D concentrations, *tfdA*, *tnpA, korB* relative abundances and α-diversity between niches and treatments, T-test, ANOVA and post-hoc Tukey tests were used in case of parametric data and Kruskal-Wallis and Wilcox’s *post hoc* tests in case of non-parametric data. Regarding β-diversity, differences between treatments and niches were compared using permutation tests (Adonis, 10,000 runs, R-package vegan; Oksanen et al., 2022) and pairwise-Adonis (Martinez Arbizu, 2020).

## 3 Results

### 3.1 2,4-D degradation in the rice paddy microcosms

**Figure 1:**
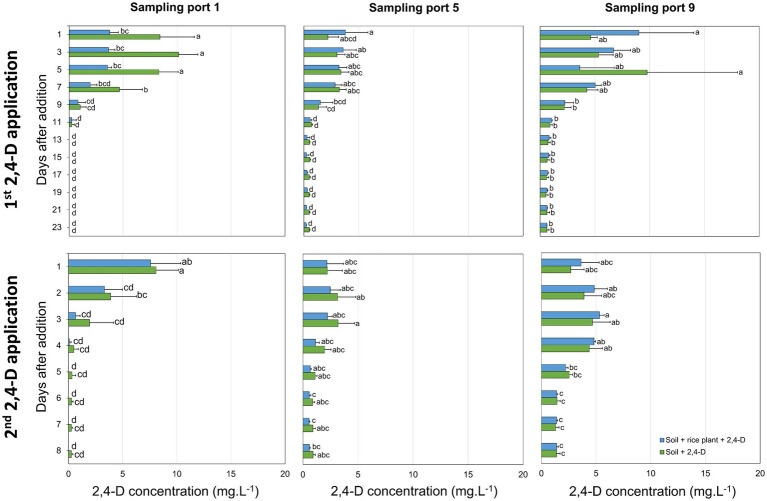
Time-lapse 2,4-D concentrations in the rice paddy microcosms after the first and second application of 2,4-D. 2,4-D concentrations in the “Soil + rice plant +2,4-D” (blue) and “Soil +2,4-D” (green) treatments were determined at sampling ports 1, 5, and 9 (from top to the bottom of the cylindric microcosms). Reported values are the average of three replicates with standard deviation. Different letters indicate values that are significantly different between the treatments (ANOVA, *p* < 0.05).

2,4-D was added equally over the length of the cylindric microcosm to provide each niche and its microbiota with similar 2,4-D concentrations. No difference in growth and morphological appearance of the rice plant was observed in 2,4-D treated microcosms and microcosms that did not receive 2,4-D, neither after the first nor after the second application of 2,4-D. After the first application of 2,4-D, 2,4-D concentrations at all three sampling ports decreased with time, indicating that 2,4-D degradation occurred at all depths of the microcosm independent of the presence of the rice plant (Figure 1). At day 13 after 2,4-D application, in both the “Soil + rice plant +2,4-D” and “Soil +2,4-D” treatments, 2,4-D was not detected anymore at sampling port 1 while still being present at low concentrations at sampling ports 5 (0.28 ± 0.25 mg.L^−1^ and 0.53 ± 0.03 mg.L^−1^, respectively) and 9 (0.82 ± 0.15 mg.L^−1^ and 0.71 ± 0.21 mg.L^−1^, respectively) indicating slower removal at deeper layers. After the second 2,4-D application, 2,4-D was removed faster compared to after the first application. Concentrations of 2,4-D were already below the detection limit at sampling port 1 at days 4 to 5 after 2,4-D application and at sampling port 5 at days 5 to 6 after 2,4-D application. Again, 2,4-D removal at deeper layers appeared slower since 2,4-D was still detected, albeit at very low concentrations, at sampling port 9 until day 8 after application (Figure 1).

### 3.2 Distribution of the 2,4-D catabolic *tfdA* gene(s) among the different niches in the rice paddy microcosms

The presence of class I, II, and III *tfdA* in the niches of the paddy rice microcosms was first examined by end-point PCR. Only the class I *tfdA* variant was detected (see Supplementary Figure S2). The class I *tfdA* was detected in most niches including the bulk soil and its detection did not depend on the presence of the rice plant. The intensities of the PCR signals using template DNA from the surface water and surface soil were clearly stronger than those obtained with DNA from the rhizosphere, root surface, and bulk soil niches. While the visual intensity of the band in endpoint PCR is only a semi-quantitative measure, it indicated a higher abundance of class I *tfdA* in surface water and surface soil compared to the other niches. Moreover, in the surface water and surface soil niches, PCR signals were stronger for the 2,4-D-treated systems compared to the non-treated systems, indicating that 2,4-D amendment resulted into a proliferation of the class I *tfdA* catabolic genotype in those two niches.

**Figure 2:**
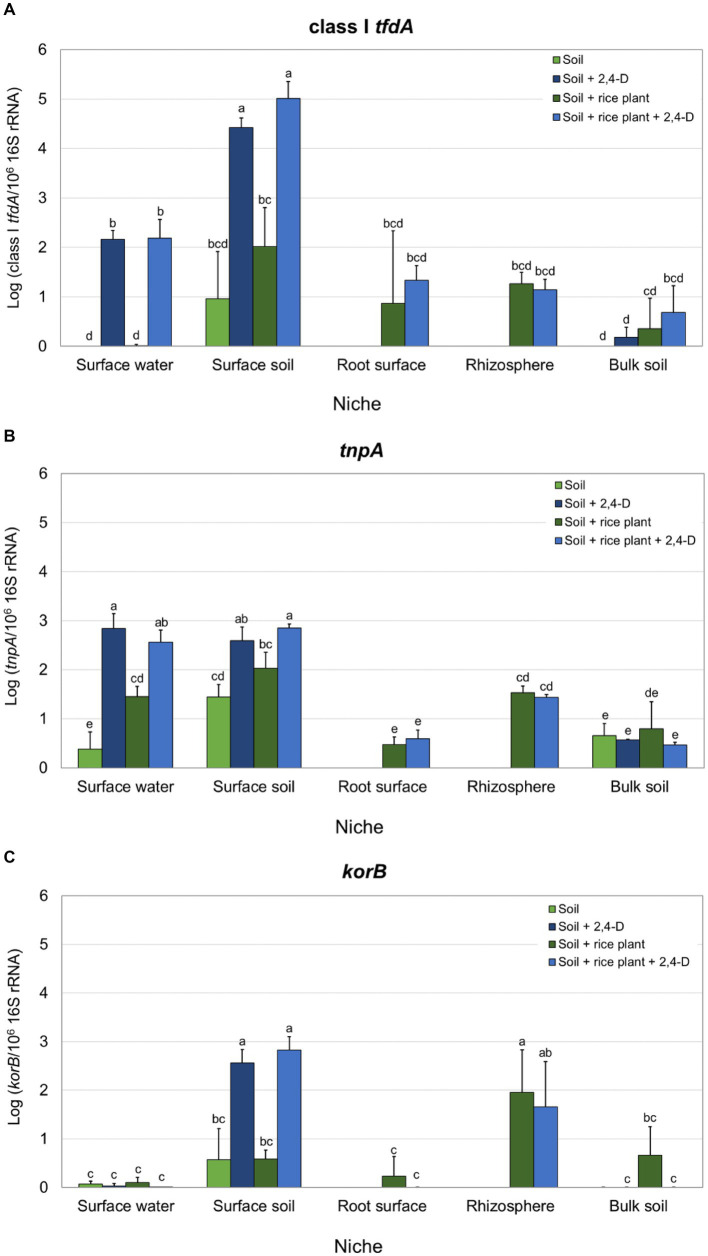
Relative abundances of class I *tfdA* **(A)**, IS*1071* (*tnpA*) **(B)** and IncP-1 plasmids (*korB*) **(C)** in the different treatments for each niche as determined by qPCR. Relative abundances are expressed as the log of the ratios of the number of target gene copies over the number of 10^6^ 16S rRNA gene copies. Reported values are the average of three replicates with standard deviation. Different letters indicate values that are significantly different over all niches (ANOVA, *p* < 0.05).

Since only class I *tfdA* was detected, quantification of *tfdA* by qPCR was only performed for class I *tfdA*. Class I *tfdA* was detected by qPCR in most of the niches in all treatments except in the surface water and bulk soil of the “Soil” treatment and the surface water of the “Soil + rice plant” treatment. Moreover, relative abundances of *tfdA* were very low in the bulk soil niche independent of the treatment. In 2,4-D amended microcosms, surface water and surface soil appear more favorable for *tfd* governed 2,4-D biodegradation since those niches showed a significantly higher relative abundance of *tfdA* compared to other niches (Turkey’s *post hoc*, *p* < 0.05) (see Supplementary Figure S3A). Moreover, 2,4-D application caused a clear and significant increase in class I *tfdA* relative abundance in surface water (relative abundances were 2.16 ± 0.18 in the “Soil +2,4-D” treatment and 2.18 ± 0.38 in the “Soil + rice plant +2,4-D” treatment while *tfdA* was not detected in the “Soil” treatment and its relative abundance was 0.01 ± 0.02 in the “Soil + rice plant” treatment) as well as in surface soil (relative abundances were 4.42 ± 0.19 in the “Soil +2,4-D” treatment and 5.01 ± 0.35 in the “Soil + rice plant +2,4-D” treatment while 0.96 ± 0.96 in the “Soil” treatment and 2.02 ± 0.78 in the “Soil + rice plant” treatment) (Figure 2A). There were no significant differences in class I *tfdA* relative abundances between 2,4-D amended and non-amended systems in the rhizosphere and root surface niches (Figure 2A).

### 3.3 Distribution of IncP-1 plasmids and IS*1071* among the different niches in the rice paddy microcosms

The relative abundances of *korB* and *tnpA* as genetic markers for, respectively, IncP-1 plasmids and IS*1071*, previously recognized as MGEs carrying the *tfd* genes (Nguyen et al., 2019), were determined in the different rice paddy treatments and niches. A significantly higher relative abundance of IS*1071* was recorded in both the surface water and surface soil and of IncP-1 in surface soil compared to the other niches in 2,4-D amended microcosms (ANOVA, *p* < 0.05) (see Supplementary Figures S3B,C). Moreover, adding 2,4-D significantly increased the relative abundance of IS*1071* in surface water (relative abundance in the “Soil +2,4-D” treatment was 7.5 fold higher than in the “Soil” treatment while it was 1.8 fold higher in the “Soil + rice plant +2,4-D” treatment compared to the “Soil + rice plant” treatment) and in surface soil (relative abundances in the “Soil +2,4-D” treatment was 1.8 fold higher than in the “Soil” treatment while it was 1.4 fold higher in the “Soil + rice plant +2,4-D” treatment compared to the “Soil + rice plant” treatment) (Figure 2B). Similarly, 2,4-D addition increased the IncP-1 relative abundance in surface soil (relative abundances in the “Soil +2,4-D” treatment was 4.5 fold higher than in the “Soil” treatment and 4.9 fold higher in the “Soil + rice plant +2,4-D” treatment compared to the “Soil + rice plant” treatment) (Figure 2C). Higher relative abundances of IncP- 1 and IS*1071* were also found in the rhizosphere compared to the root surface and bulk soil but without being affected by the addition of 2,4-D. As for *tfdA*, the presence of the rice plant did not show any effects on IS*1071* and IncP-1 plasmids relative abundances, except in surface water where the relative abundance of IS*1071* in the “Soil + rice plant” treatment, was higher than in the “Soil” treatment (Figures 2B,C).

### 3.4 Bacterial community structure in the niches of the rice paddy microcosms

#### 3.4.1 α-diversity profile of the bacterial community composition across niches and treatments

**Figure 3:**
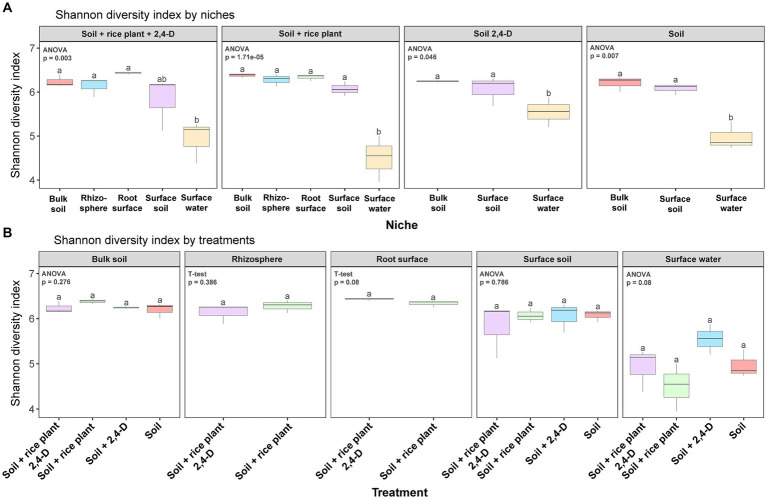
Box plots of α-diversity (Shannon diversity) in the different niches for each treatment **(A)** and in the different treatments for each niche **(B)** in the rice paddy microcosms. Box plot represents: whiskers = minimum and maximum values, the box = the range between 1st (lower) and 3rd (upper) quartiles, horizontal bold line = median. Different letters indicate values that are significantly different between the niches in one treatment in **(A)** and between the treatments in one niche in **(B)** (ANOVA/*T*-test, *p* < 0.05).

Following quality control and processing, a total of 7,237 ASVs (defined with a sequence identity of 99%) were identified across all soil samples. In terms of α-diversity, the Shannon diversity index, ASV richness, and phylogenetic diversity were lower in the surface water compared to all other niches, i.e., surface soil, rhizosphere, root surface, and bulk soil across all treatments (Tukey’s *post hoc*, *p* < 0.05) (see Figure 3A; Supplementary Figures S4A, S5A). Moreover, surface water was the only niche showing some significant differences in α-diversity between treatments (ANOVA, *p* < 0.05) (see Figure 3B; Supplementary Figures S4B, S5B). Specifically, the α-diversity of the bacterial community in the “Soil +2,4-D” treatment was higher compared to all other treatments (Tukey’s *post hoc*, *p* < 0.05).

#### 3.4.2 β-diversity profile of the bacterial community composition across niches and treatments

**Figure 4:**
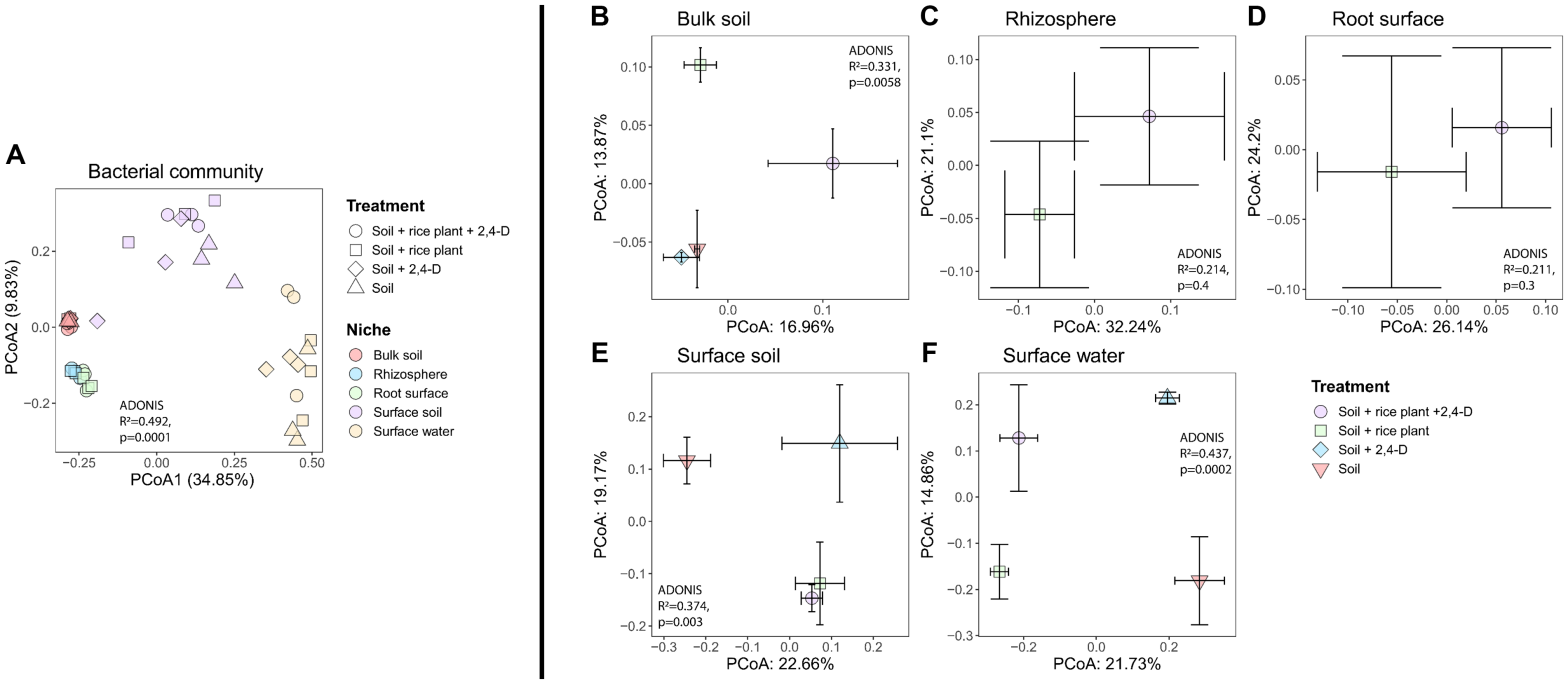
Variation in bacterial community structure based on Bray–Curtis dissimilarity, depicted by PCoA in **(A)** all niches and treatments, **(B)** bulk soil, **(C)** rhizosphere, **(D)** root surface, **(E)** surface soil, **(F)** surface water. The bacterial community structure differed significantly according to the niche **(A)** while a significant impact of treatment on community composition was observed for the bulk soil, surface soil and surface water **(B,E,F)**, as determined by PERMANOVA, *p* < 0.001. Centroids for each treatment are shown along with their standard errors (error bars).

In order to assess the structure of soil bacterial communities across niches and treatments, we computed Bray-Curtis dissimilarities. Bacterial community compositions clustered into four distinct clusters according to the niche (Figure 4A, Adonis niches, *R*^2^ = 0.492, *p* = 0.0001), with bacterial communities in (i) bulk soil, (ii) surface soil and (iii) surface water significantly differed from each other (pairwise-Adonis, *p* < 0.05), while the (iv) root surface and rhizosphere communities clustered together (pairwise-Adonis, *p* > 0.05). SIMPER analysis explained the observed differences between niches by a wide variety of bacterial taxa detailed in Supplementary Table S2. Especially, notable were the higher abundances of the majority of phototrophic Cyanobacterial taxa in the surface soil and especially the surface water compared to the other niches as also shown by the taxa bar plots composed at phylum level (see Supplementary Figure S6). While rhizosphere, root surface, and bulk soil had comparable compositions, surface water, and surface soil showed their unique phyla such as *Chlamydae, Gemmatimodetes, Verrucomicrobia,* and *Cyanobacteria* with *Cyanobacteria* occupying quite high proportions in surface water and surface soil. Furthermore, a much higher number of cyanobacterial ASVs were found in the surface water (28 ASVs) and surface soil (16 ASVs) compared to the bulk soil (3 ASVs). These ASVs belonged also to four classes of Cyanobacteria (4C, ML635J-21, *Nostocophycideae*, *Oscillatoriophycideae*) in the surface water and surface soil, while only to two classes (*Oscillatoriophycideae* and ML635J-21) in the bulk soil. The taxa bar plot at phylum level further showed that Proteobacteria were present in all niches with the highest relative abundances in the oxic niches (surface water, surface soil, rhizosphere, and root surface), independent of the treatment.

To investigate the effect of 2,4-D addition and the presence of the rice plant on the bacterial community structure in the various niches, we evaluated the treatment effect using the Bray-Curtis metric for each individual niche. Effects of the treatment indeed varied according to the niche (Figures 4B–F), with only significant effects for the bulk soil (Figure 4B, Adonis treatment, *R*^2^ = 0.331, *p* = 0.0058), surface soil (Figure 4E, Adonis treatment, *R*^2^ = 0.374, *p* = 0.003), and surface water (Figure 4F, Adonis treatment, *R*^2^ = 0.437, *p* = 0.0002). In case of the bulk soil, the community structures of the “Soil +2,4-D” and the “Soil” treatments clustered together (pairwise-Adonis, *p* > 0.05) but they both significantly differed from the “Soil + rice plant +2,4-D” and the “Soil + rice plant” treatments (pairwise-Adonis, *p* < 0.05). However, the “Soil + rice plant +2,4-D” treatment did not differ significantly from the “Soil + rice plant” treatment (pairwise-Adonis, *p* > 0.05), indicating that mainly the presence of the rice plant and not the addition of 2,4-D drove the alteration of the community composition in the bulk soil. Multiple bacterial taxa explained the shifts between the different treatments in the bulk soil (see Supplementary Table S3A). In case of the surface soil, the “Soil + rice plant +2,4-D” and “Soil + rice plant” treatments showed similar bacterial compositions (pairwise-Adonis, *p* > 0.05), while they differed significantly from the “Soil” treatment (pairwise-Adonis, *p* < 0.05) explained by a multiplicity of bacterial taxa using SIMPER (see Supplementary Table S3B). Interestingly, in this particular niche, the “Soil +2,4-D” treatment did not exhibit significant differences compared to “Soil + rice plant +2,4-D,” “Soil + rice plant,” and the “Soil” treatments (pairwise-Adonis, *p* > 0.05). The only niche where an effect of 2,4-D was noted was the surface water. Indeed, the bacterial community composition in the “Soil +2,4-D” treatment significantly differed from that of the “Soil” treatment (pairwise-Adonis, *p* < 0.05), indicating that 2,4-D addition altered the bacterial community structure in the surface water. Taxa that increased in relative abundances when 2,4-D was added, were ASVs belonging to the families *Oxalobacteraceae*, *Hyphomicrobiaceae*, *Rickettsiaceae*, *Comamonadaceae*, *Xanthomonadaceae*, *Chitinophagaceae* and order *Bacteroidales*, *Rhizobiales*, and KD8-87 (see Supplementary Table S3C). Interestingly, the surface water community in the “Soil + rice plant +2,4-D” and “Soil + rice plant” treatments, despite differing from the “Soil +2,4-D” (pairwise-Adonis, *p* < 0.05) and the “Soil” treatments (pairwise-Adonis, *p* < 0.05), demonstrated similar community structures (pairwise-Adonis, *p* > 0.05), indicating a diminishing impact of 2,4-D addition in the surface water when the rice plant was present. We conclude from the β-diversity analysis, that the rice plant was the main factor defining the bacterial communities in surface water, surface soil, root surface, rhizosphere, and bulk soil, with limited effects of 2,4-D application except in case of the surface water when the rice plant was absent.

### 3.5 α-diversity and β-diversity profiles of the β-proteobacterial bacterial community composition across niches and treatments

**Figure 5:**
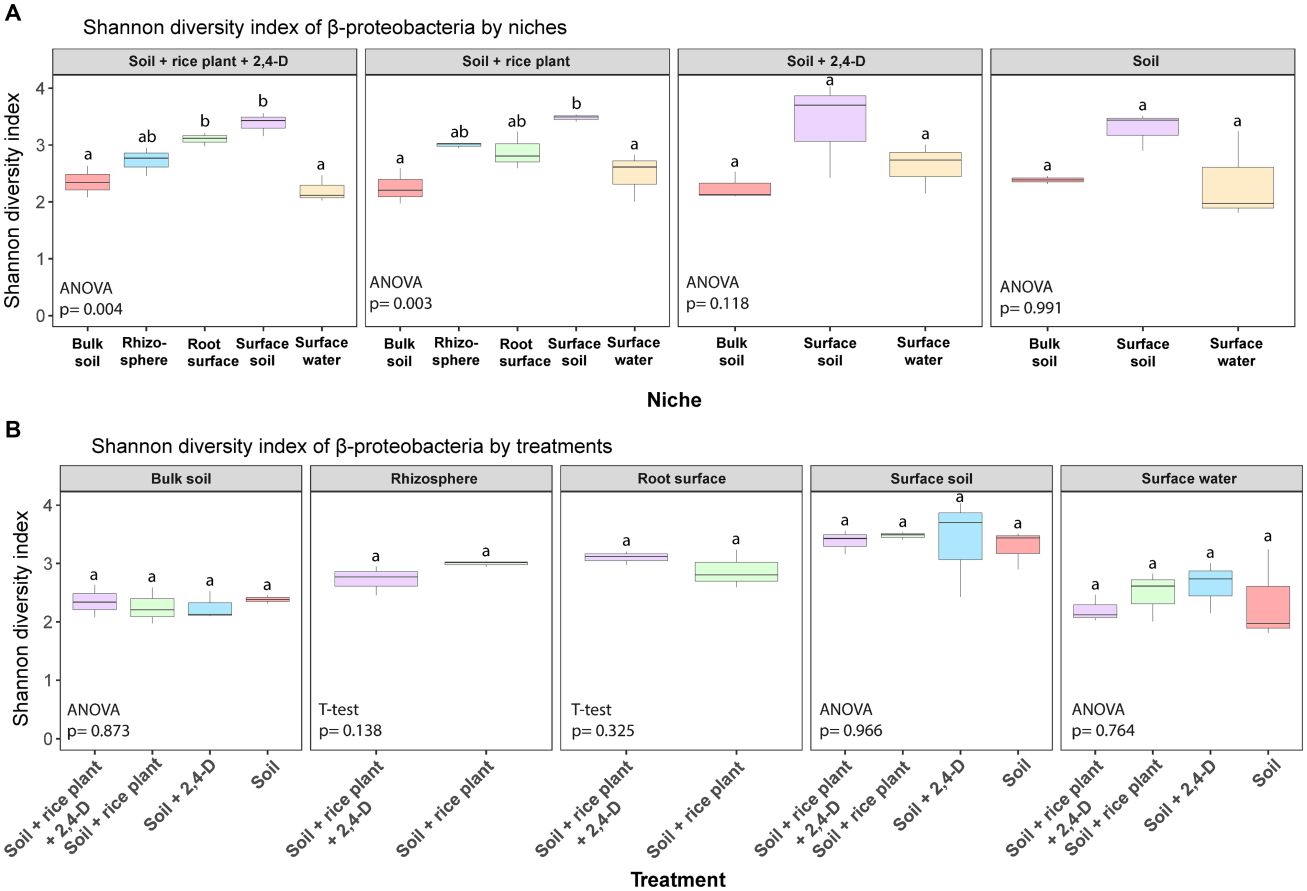
Box plots of α-diversity (Shannon diversity) of β-proteobacteria in the different niches for each treatment **(A)** and in the different treatments for each niche **(B)** in the rice paddy microcosms. Box plot represents: whiskers = minimum and maximum values, the box = the range between 1st (lower) and 3rd (upper) quartiles, horizontal bold line = median. Different letters indicate values that are significantly different between the niches in one treatment in **(A)** and between the treatments in one niche in **(B)** (ANOVA/*T*-test, *p* < 0.05).

2,4-D catabolic bacteria isolated from 2,4-D treated soils and containing *tfd* genes including the class I *tfdA* gene, are almost exclusively β-proteobacterial genera (Amy et al., 1985). Nguyen et al. (2019) reported that all 2,4-D catabolic bacterial strains isolated from rice fields with a history of 2,4-D application in Tien Giang province and Soc Trang province (where our soil was collected although in a different field) were β-proteobacteria. Therefore, we examined the differences in β-proteobacterial community structure between niches and the treatments focusing on differences between 2,4-treated systems and non-treated systems. Our findings demonstrated distinct α-diversity profiles of the β-proteobacteria across niches for each treatment (ANOVA, *p* < 0.05) (see Figure 5A; Supplementary Figures S7A, S8A) but not across treatment within a niche (see Figure 5B; Supplementary Figures S7B, S8B). The α-diversity of the β-proteobacteria communities in surface soil and surface water were generally higher compared to other niches, particularly in terms of ASV richness and phylogenetic diversity (Tukey’s *post hoc*, *p* < 0.05) (see Supplementary Figures S7A, S8A). Furthermore, in line with the α-diversity of the total bacterial communities, only the β-proteobacterial community in surface water showed significant differences among treatments (ANOVA, *p* < 0.05) (see Supplementary Figures S7B, S8B). Moreover, as for the total bacterial community, the “Soil +2,4-D” treatment showed a higher α-diversity compared to the other treatments (Tukey’s *post hoc*, *p* < 0.05) suggesting an impact of 2,4-D addition. However, the presence of plants diminished the impact of 2,4-D as the “Soil + rice plant +2,4-D” treatment showed a similar α-diversity as the “Soil” treatment (Tukey’s *post hoc*, *p* > 0.05).

**Figure 6:**
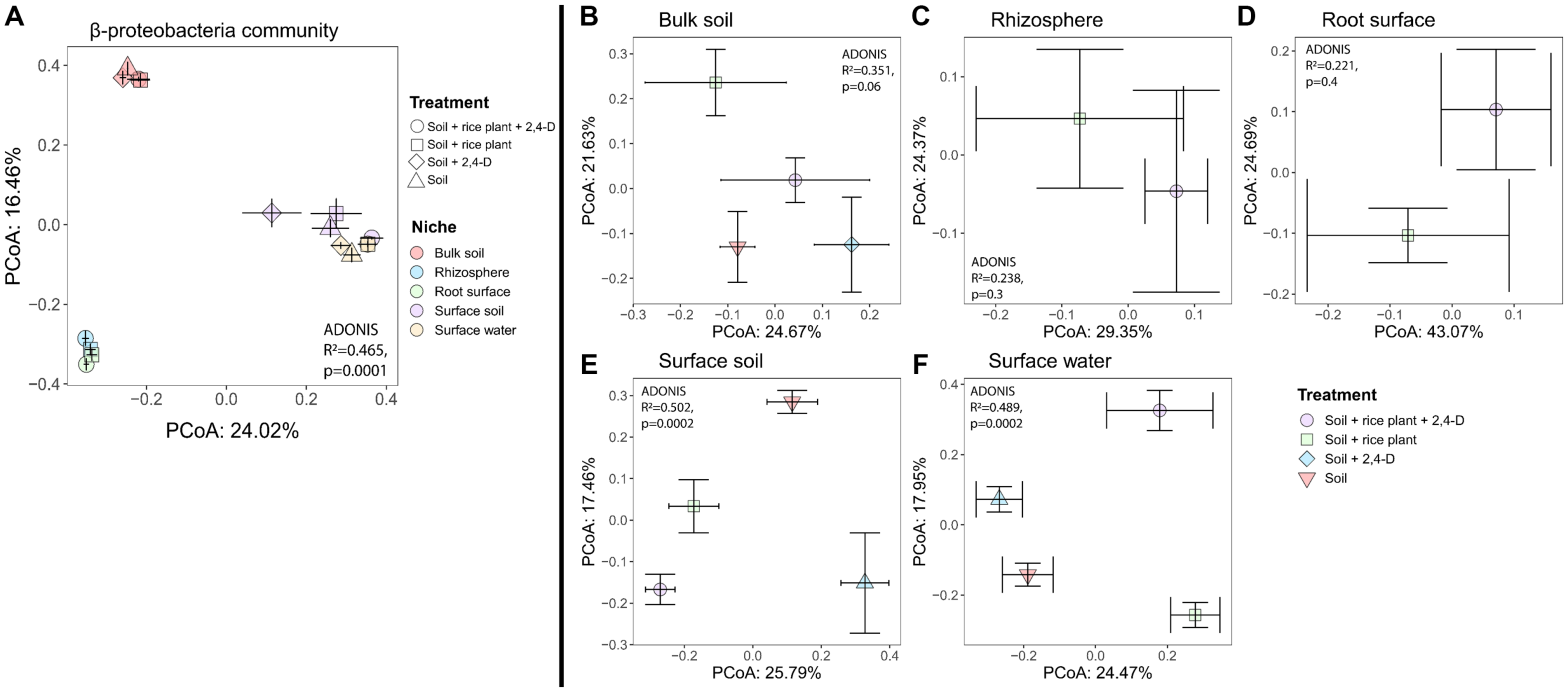
Variation in the β-proteobacterial community structure based on Bray–Curtis dissimilarity, depicted by PCoA in **(A)** all niches and treatments, **(B)** bulk soil, **(C)** rhizosphere, **(D)** root surface, **(E)** surface soil, **(F)** surface water. Based on niche **(A)**, the β-proteobacterial bacterial community clustered in three groups, i.e., (i) bulk soil, (ii) surface soil and surface water, and (iii) the root surface and rhizosphere. Impact of the treatment was significantly observed in the surface soil and surface water **(E,F)**, as determined by PERMANOVA, *p* < 0.0001. Centroids for each treatment are shown along with their standard errors (error bars).

As for the total community structure, niche effects based on Bray-Curtis were also prominent for the β-proteobacterial community (Figure 6A, Adonis niches, *R*^2^ = 0.465, *p* = 0.0001). The β-proteobacteria community clustered into three distinct groups (pairwise-Adonis, *p* < 0.05) based on the niches they inhabited, i.e., (i) bulk soil, (ii) surface soil and surface water, and (iii) the root surface and rhizosphere. When we examined the treatment effect using the Bray-Curtis metric for each individual niche, no effect was observed for the bulk soil, rhizosphere and root surface (Figures 6B–D). However, treatment effects were observed for the surface soil (Figure 6E, Adonis treatment, *R*^2^ = 0.502, *p* = 0.0002) and the surface water (Figure 6F, Adonis treatment, *R*^2^ = 0.489, *p* = 0.0002). In these niches, the β-proteobacterial community structures exhibited significant differences between each of the four treatments (pairwise-Adonis, *p* < 0.05) (Figures 6E,F) suggesting that the addition of 2,4-D and the presence of a rice plant had an impact on the β-proteobacterial community structure in surface soil and surface water. SIMPER analysis showed that in the surface soil, ASVs affiliated with the genera *Acidovorax* and *Georgfuchsia* exhibited a clear higher relative abundance in the “Soil +2,4-D” treatment compared to the “Soil” treatment (see Supplementary Table S4A). In contrast, ASVs associated with the families *Comamonadaceae* (but different from *Acidovorax*) and *Rhodocyclaceae* showed lower relative abundances. Comparing the “Soil + rice plant +2,4-D” treatment with the “Soil + rice plant” treatment, ASVs from the families *Comamonadaceae, Oxalobacteraceae, Rhodocyclaceae*, and *Hydrogenophilaceae* were more relatively abundant in the “Soil + rice plant +2,4-D” treatment whereas one ASV from the family *Oxalobacteraceae* was less abundant showing that the plant, as in the case of the total community, could change effects of 2,4-D on the β-proteobacteria community composition. Making pairwise comparisons between the different treatments, overall the plant seems to stimulate ASVs from the families *Comamonadaceae* and *Oxalobacteraceae* while ASVs from the genera *Acidovorax* and *Georgfuchsia* were stimulated when 2,4-D was added. Interestingly, as indicated by the comparison between the “Soil + rice plant +2,4-D” treatment and the “Soil +2,4-D” treatment, the presence of the plant decreased the *Acidovorax* and *Georgfuchsia* relative abundances. ASVs belonging to the β-proteobacterial family *Burkholderiaceae* like *Burkholderia* sp., *Cupriavidus* sp., and *Ralstonia* sp. previously associated with *tfd-*mediated 2,4-D biodegradation, were not detected (see Supplementary Table S4A).

Also in surface water, comparing the “Soil +2,4-D” treatment to the “Soil” treatment showed the higher relative abundances of ASVs associated with the genus *Acidovorax* but also with ASVs of the family *Rhodocyclaceae* in the “Soil +2,4-D” treatment. In contrast to the surface soil, no β-proteobacterial taxa showed a significant decrease in abundance between the two treatments. Comparing the “Soil + rice plants +2,4-D” treatment with the “Soil + rice plant” treatment, ASVs that were significantly higher in the “Soil + rice plant +2,4-D” treatment belonged to the genera *Acidovorax, Pseudacidovorax, Herbaspirillum,* and *Denitromonas* from the families *Comamonadaceae, Oxalobacteraceae,* and *Rhodocyclaceae* and we did not find any β-proteobacterial ASVs that were less abundant. Looking at the effect of the presence of the plant, overall, unidentified β-proteobacteria seemed to be stimulated when the plant was present but interestingly only when 2,4-D was added while these organisms were not stimulated when 2,4-D was added in systems without the plant. Taxa belonging to the family *Comamonadaceae, Oxalobacteraceae, Rhodocyclaceae,* and the genera *Georgfuchsia, Nitrosomonas,* and *Leptothrix* were overall less abundant when the rice plant was present. Also, here this seems to be counteracted by adding 2,4-D. As for the surface soil, ASVs belonging to the β-proteobacterial family *Burkholderiaceae* like *Burkholderia* sp., *Cupriavidus* sp., and *Ralstonia* sp. previously associated with *tfd-*mediated 2,4-D biodegradation, were not detected (see Supplementary Table S4B).

## 4 Discussion

### 4.1 2,4-D biodegradation in the rice paddy ecosystem

By using dedicated microcosms, this study is the first that compartment-specifically analyzes the biodegradation of a pesticide in the rice paddy ecosystem focusing on *tfd* governed 2,4-D biodegradation. The findings indicate that 2,4-D is rapidly degraded in the system along the whole length of the soil column. Since this study in the first place aimed to examine the niches of *tfd* governed 2,4-D biodegradation, sterile soil systems were not included. Nevertheless, the slower removal of 2,4-D observed in the deeper layers compared to the higher layers suggests the contribution of biological degradation apart from sorption, light-induced and chemical degradation processes. Moreover, we expect limited adsorption in the soil as the soil pH was around 4.3 while the pKa value of 2,4-D is 2.8 (Islam et al., 2018). The rice paddy is a complex environment containing adjacently located aerobic and anaerobic compartments/niches. Although the bulk soil is dominantly anaerobic, there are aerobic niches, i.e., the surface water, surface soil, rhizosphere, and root surface, which can provide oxygen for aerobic microbial processes (Liesack et al., 2000). The fast 2,4-D removal in all layers, suggests that both aerobic and anaerobic biodegradation takes place. No effect of the plant on 2,4-D removal was observed but likely the low spatial resolution of the sampling approach does not allow to determine local 2,4-D concentrations at the root surface/rhizosphere compartment and hence to directly determine root surface/rhizosphere effects. 2,4-D biodegradation has been shown before to occur under aerobic as well as anaerobic conditions. The latter includes biodegradation under methanogenic conditions in rice paddy soils (Yang et al., 2017) and in aquifers (Gibson and Suflita, 1986), and under sulfate-reducing conditions in mineral clayish agricultural soil (Robles-González et al., 2006). Rates of 2,4-D biodegradation are expected to be faster under oxic conditions (t_1/2_ = 6.2 to 15 days) as compared to anoxic conditions (t_1/2_ = 41–333 days) (Environmental Protection Agency, 2005) while oxygen concentrations in surface soil and surface water are expected to be higher than at the root surface and in the rhizosphere (Li et al., 2014).

No effect of 2,4-D on the rice plant was observed. While 2,4-D is a systemic herbicide leaving cereals relatively unaffected, this observation is in contrast with a rice paddy field study in which young rice seedlings showed abnormal symptoms such as a swollen base and stubby roots, after applying 2,4-D. On the other hand, within two or three weeks of 2,4-D application, the plants recovered and grew normal root systems (Ryker and Brown, 1947). Interestingly, phytotoxicity effects of the 2,4-D related phenoxyacetic acid herbicide 2-methyl-4-chlorophenoxyacetic acid (MCPA), whose activity, like 2,4-D, mimics the action of the plant growth hormone auxin, was also observed on *Sorghum saccharatum* but its effect was diminished by native MCPA-degrading bacteria in the soil (Mierzejewska et al., 2018). Alleviation or mitigation of phytotoxicity of a pesticide in soil has also been demonstrated for different plants when bacteria able to degrade the pesticide were inoculated in the soil including systems that received 2,4-D and inoculated with 2,4-D catabolic bacteria (Vaishampayan et al., 2007; Xia et al., 2017; Jiang et al., 2023; Wang et al., 2023). Therefore, the observed 2,4-D biodegradation might have contributed to alleviating any phytotoxicity in our paddy soil microcosm experiment.

### 4.2 Relative abundances of *tfdA* appoint surface water and surface soil as the favorable niches for aerobic 2,4-D biodegradation in rice paddy ecosystems

*tfdA*, *tfdAα,* and *cadA* are catabolic genes encoding the initial oxygenation of 2,4-D in bacteria and represent different iso-functional 2,4-D catabolic pathways (Kitagawa and Kamagata, 2014). Since *tfdA* is prevalent in fast 2,4-D degraders isolated from 2,4-D treated soils (Amy et al., 1985) while *tfdAα* and *cadA* are predominantly found in isolates derived from non-contaminated pristine environments (Kamagata et al., 1997; Kitagawa et al., 2002) and 2,4-D treated rice paddy fields in the Mekong delta in Vietnam were previously shown to contain *tfdA* carrying bacteria (Nguyen et al., 2019), we focused on *tfdA*. Sequence-wise, three subtypes of *tfdA* can be recognized, i.e., class I, II, and III with DNA sequence similarities between the three classes ranging from 76–92% (Baelum et al., 2010). Our end-point PCR indicated the exclusive presence of class I *tfdA* in the samples which matched the study of Nguyen et al. (2019) in which 2,4-D catabolic bacteria were isolated, exclusively containing the class I *tfdA* gene, from rice paddy fields that were located near to those sampled in the current study. These observations suggest that the organisms harboring class I *tfdA* were the most abundant *tfd* containing 2,4-D catabolic bacteria contributing to 2,4-D biodegradation in the collected rice paddy soil samples. This differs from observations by Baelum and Jacobsen (2009) who reported that adding 2,4-D in several soils was accompanied with the growth of organisms carrying class I or class III *tfdA* with a higher proportion of class III *tfdA*. Consequently, we employed only class I *tfdA* in quantitative PCR to assess the aerobic 2,4-D biodegradation capacity in the microcosm. In 2,4-D treated microcosms, class I *tfdA* relative abundances in surface water and surface soil were 2–4-fold higher than in the root surface and rhizosphere niches, and 3–25-fold higher than in bulk soil. Moreover, the abundances of class I *tfdA* in surface water and surface soil increased 2–170-fold in response to 2,4-D application compared to these niches in the non-treated microcosms. These findings agree with the observed faster rates of 2,4-D disappearance in the upper layers compared to the deeper layers of the microcosms. It suggests that 2,4-D degradation occurred dominantly in the surface soil and surface water concomitant with an increase in 2,4-D degraders containing the *tfdA* gene. Previous studies reported that 2,4-D addition increased the density of 2,4-D degraders, *tfdA* transcription, and *tfdA* abundance in ponds (de Lipthay et al., 2002) and in agricultural soil (Baelum et al., 2008). In contrast to the surface water and surface soil, no response of class I *tfdA* on the addition of 2,4-D occurred in the bulk soil, rhizosphere and root surface niches. The difference can be explained by the differences in oxygen levels between water-soil interface, the bulk soil and the rhizosphere interface, taking into account that oxygen is a main determinant of 2,4-D biodegradation rate (Environmental Protection Agency, 2005). The oxygen in the water-soil interface derives from the atmosphere through diffusion but also from photosynthetic organisms like algae and cyanobacteria which were indeed present at higher relative abundances in the surface soil and surface water compared to other compartments. The presence of photosynthetic organisms can make oxygen over 200% saturation or push the oxic-anoxic interface down to a few mm depth during the day (Roger et al., 1993; Liesack et al., 2000). Nevertheless, rice paddies are characterized by a steep vertical oxygen concentration gradient showing complete depletion of oxygen in the bulk soil from around 2 to 10 mm depth (Lüdemann et al., 2000; Lee et al., 2015). That this was also the case in our microcosm set up, is suggested by the observation of brownish iron plaque till around 5 mm depth due to the oxidation of ferrous iron by oxygen to form brownish ferric ion oxides that locally precipitate (see Supplementary Figure S9). Oxygen concentrations in the rhizosphere interface are expected to be much lower than in the water-soil interface because it only derives from diffusive transport from the aerenchymatous tissue (Liesack et al., 2000). In addition, aerobic 2,4-D biodegradation at the level of the root surface has to compete for the limited oxygen with other aerobic microbial processes such as nitrification, methane oxidation, and the oxidation of root exudates (Roger et al., 1993; Van Bodegom et al., 2001; Guo et al., 2021), which might have limited the net-growth of root surface/rhizosphere 2,4-D catabolic bacteria without excluding their contribution to 2,4-D biodegradation. That oxygen was released at the level of the root surface and in the rhizosphere was, as in the case of the surface soil, suggested by the formation of brownish iron plaque on and around the root surface (see Supplementary Figure S9) (Chen et al., 2008). The increase in relative abundance of *tfdA* in surface water and surface soil after 2,4-D application, suggests that these two niches provided favorable conditions for aerobic 2,4-D biodegradation. Moreover, it suggests the growth of the *tfdA*-carrying bacteria which is only possible when they contain a complete *tfd* gene module while implicating that 2,4-D was mineralized.

Besides the relative abundances of class I *tfdA*, also the relative abundance of IS*1071* increased in surface soil and surface water in response to 2,4-D application, while an increase in relative abundance of IncP-1 plasmids upon 2,4-D application, was observed in surface soil. High relative abundances of these two MGEs were also detected in the rhizosphere compared to the bulk soil, but independent of the addition of 2,4-D. The similar behavior of these two MGEs and class I *tfdA* in the same niches showed that they are likely linked and suggests that *tfdA* is carried by IS*1071* and IncP-1 plasmids in the examined soil system. This agrees with previous studies showing that xenobiotic catabolic gene clusters including 2,4-D catabolic gene clusters containing *tfdA*, are often carried by IncP-1 plasmids. Moreover, on IncP-1 plasmids, they are often bordered by IS*1071* within a composite transposon configuration. MGEs such as IncP-1 and IS*1071* are therefore considered major vehicles of the evolution and distribution of xenobiotic catabolic genes (Top and Springael, 2003; Nojiri et al., 2004; Dennis, 2005) as recently confirmed by targeted metagenomics (Dunon et al., 2018, 2022). Furthermore, the carriage of the *tfd*-gene cluster (including *tfdA*) by IncP-1 plasmids and their flankage by IS*1071* was also shown for the 2,4-D catabolic bacteria isolated from rice paddy fields in the Mekong Delta, Vietnam in the study of Nguyen et al. (2019). The carriage of the *tfd*-genes by MGEs like IS*1071* and IncP-1 plasmids make the catabolic genes prone to HGT and hence could have contributed to the increase of the *tfdA* relative abundances and the capacity of aerobic 2,4-D degradation in the surface soil and surface water compartments.

### 4.3 Community composition is niche-dependent and affected by an interplay between the plant and 2,4-D addition

The analysis of the bacterial communities revealed distinct compositions within each niche, with the exception of the root surface and the rhizosphere which exhibited a similar structure. Other studies also examined community composition differences between spatially separated compartments in rice paddy soils. While some focused on the vertical oxygen gradient from surface to deeper layers in the bulk soil without considering the plant (Lüdemann et al., 2000; Noll et al., 2005), others addressed the rhizosphere community (Edwards et al., 2015; Li et al., 2019). However, no studies included the surface water and our study is the first that considers all compartments except for the endosphere. The community composition of the surface water and surface soil was more similar to each other than to those of other compartments but clearly differed. Oxic layers in paddy soil vary from 2 to 20 mm depending on the oxygen diffusion from the water surface and the concentration of organic matter in the soil (Roger, 1996). In our study, the surface soil compartment was defined by the first 5 mm of the top soil. While the appearance of iron plaque seems to support this (see above), the depth where oxygen becomes fully depleted is not clearly delineated, and therefore the community composition of the surface soil might have been affected by organisms of the oxic-anoxic interface or the anoxic bulk soil. On the other hand, differences in major microbial activities involving different microbial guilds in surface water and surface soil of rice paddies have been reported (Roger et al., 1993). Major activities in surface water include photosynthesis and respiration by aquatic biomass and photo-dependent biological nitrogen fixation by free-living and symbiotic cyanobacteria, while in the surface soil, key microbial activities are nitrification, methane oxidation, photodependent biological nitrogen fixation by algae and photosynthetic bacteria, denitrification, iron reduction, and methanogenesis (Watanabe and Furusaka, 1980). Furthermore, the community composition in the surface soil differed from that of the bulk soil which is explained by differences in the oxygen concentration. In our study, bulk soil samples originated from the soil from 5 mm depth and more. Lee et al. (2015) observed clear distinct communities in rice paddy soils at 1 mm resolution scale over a gradient of oxygen that drastically decreased from 8 mm depth and defining an oxic, oxic-anoxic interface and anoxic zone. Lüdemann et al. (2000) showed similar community changes along the vertical oxygen gradient in rice paddy microcosms but with the oxic-anoxic interface at 2 mm depth. Our study further aligns with other studies showing that in rice paddy soil, the rhizosphere and root surface community differed from the bulk soil community (Edwards et al., 2015). However, in contrast and despite using a similar protocol to separate both niches, in our study, the rhizosphere surface community did not differ from the root surface community. Nevertheless, this shows that plant roots can influence community composition and select for certain bacterial types (Edwards et al., 2015). Apart from the local release of oxygen, this can be due to the release of water-soluble root exudates and/or insoluble materials, lysates, dead fine roots, and gasses such as CO_2_ and ethylene, which can serve as sources of energy and nutrients (Tian et al., 2013). The importance of the plant in shaping the community was emphasized by the differences in bulk soil community structure between treatments with and without the plant indicating that the effect of the plant on the bulk soil community extends beyond the rhizosphere. Interestingly, the β-proteobacterial community, a taxon that harbors the majority of the currently known 2,4-degrading bacterial isolates carrying *tfd*, largely followed the total community composition except that the surface water and surface soil community structure showed no significant differences.

The addition of 2,4-D had only a minor impact on the community composition of both bacteria and β-proteobacteria. Indeed, 2,4-D only affected the community in the surface soil and surface water compartments. 2,4-D affected the total community in the surface water in case the rice plant was absent but not in the presence of the plant. This indicates that the plant counteracts 2,4-D effects, highlighting the prevalence of complex interactions between 2,4-D, the plant, and the microbiota. On the other hand, considering only the β-proteobacterial community, significant differences were found between each of the four treatments in both the surface water and surface soil compartments when 2,4-D had been applied. Our primary goal in analyzing community composition was to identify organisms that exhibited an increase in relative abundances in the 2,4-D treatments compared to non-treatments, suggesting a potential involvement in 2,4-D catabolism. We especially anticipated the identification of genera like *Burkholderia* sp., *Cupriavidus* sp., and *Ralstonia* sp. belonging to the β-proteobacterial family *Burkholderiaceae*. These bacteria were previously isolated from rice fields near those that delivered the soil in the current study (Nguyen et al., 2019) as well as from rice fields in Nigeria (Muhammad et al., 2023) and carried *tfdA*. The same genera were also isolated as 2,4-D degraders in a study by Macur et al. (2007) that examined the impact of 2,4-D application on community composition in upland crops. However, our community analysis did not uncover any ASVs associated with these species or their family. Instead, it identified the presence of members of another β-proteobacterial family, i.e., *Comamonadaceace*. We propose three hypotheses to explain this discrepancy. First, sampling was conducted after the 2,4-D biodegradation phase resulting in the decay and hence reduction of any 2,4-D catabolic population that had developed during the biodegradation phase. While Gonod et al. (2006) reported that 2,4-D changed the soil bacterial community structure only during the intense phase of 2,4-D degradation and rapidly returned to its initial composition when 2,4-D was depleted, the observed differences in *tfdA* relative numbers between 2,4-D treated and non-treated surface water and surface soil suggest otherwise. Second, the 2,4-D degrading population might be too sparse in relative abundance to be detected. The highest relative abundances of *tfdA* detected were 364 copies per 10^6^ 16S rRNA equivalent to around 4 in 10,000 bacterial cells. Using a sequencing depth of 25,000 16S rRNA gene reads per sample, only a few sequences could derive from 2,4-D degrading bacteria, making their detection challenging, especially in case they belong to multiple species. Third, bacteria belonging to other families, like *Comamonadaceace* performed *in situ* 2,4-D degradation in the microcosms and were the hosts of *tfdA*. An aerobic 2,4-D degrading strain, i.e., Var*iovorax koreensis*, that belongs to the *Comamonadaceace* family and that carries *tfdA* has been reported before (Tonso et al., 1995). HGT of the *tfd* gene cluster involving MGEs like IncP-1 and IS*1071*, from organisms like *Cupriavidus,* makes the catabolic machinery available to other taxa, including species belonging to the related *Comamonadaceace* taxon (Goris et al., 2002). Nevertheless, the effect of 2,4-D on community composition was most evident in the two compartments (surface water and surface layer) that showed an increase of *tfdA* relative abundances in the 2,4-D treated microcosms compared to non-treated microcosms. However, besides stimulating the growth of 2,4-D degraders (de Lipthay et al., 2002; Macur et al., 2007; Baelum et al., 2008), the introduction of 2,4-D can also affect the bacterial community composition in alternative ways. As demonstrated for other pesticides (Sebiomo et al., 2011), 2,4-D can exert direct toxic effects on sensitive populations (Breazeale and Camper, 1970). Moreover, although not visibly observed in this study, 2,4-D can impact plant physiology which might indirectly affect microbial community members, for instance, by affecting root exudate production (Balasubramanian and Rangaswami, 1973). Moreover, those direct and indirect effects on specific groups of bacteria might have further effects on the community structure by untying existing interactive networks and exchange of niches between species (Baquero et al., 2021). Those mechanisms would lead to both taxa that increase and taxa that decrease in relative abundance as observed in our study and in others (Gonod et al., 2006; Macur et al., 2007; Moretto et al., 2017; Chen et al., 2021; Han et al., 2022). However, discriminating between community changes due to the growth of 2,4-D catabolic bacteria or to toxic effects as a response to 2,4-D application, is challenging (Gonod et al., 2006; Macur et al., 2007; Moretto et al., 2017; Tu et al., 2019; Chen et al., 2021; Han et al., 2022), unless combined with efforts to isolate 2,4-D degraders or with DNA-Stable Isotope Probing (Macur et al., 2007). Finally, we cannot exclude that taxa that increased in relative abundances upon application of 2,4-D are taxa that used an alternative (unknown) pathway in biodegradation of 2,4-D, different from the *tfd* governed pathway. A next step would be to examine whether *tfd*-carrying bacteria and communities in the different compartments behave similarly upon 2,4-D application in rice paddy systems in the field. Moreover, it would be of interest to examine whether similar roles for the different compartments exist for 2,4-D degrading bacteria that depend on alternative catabolic pathways and for biodegradation of other pesticides. Moreover, isolation and characterization of 2,4-D catabolic bacteria from the different individual compartments might shed further light on the mechanisms underlying 2,4-D degradation in the paddy rice field system and the role of its different niches in 2,4-D degradation.

## Data Availability

The data presented in the study are deposited in the Sequence Read Archive (SRA) repository (NCBI), accession number PRJNA1145605. The data can be accessed via the link: https://www.ncbi.nlm.nih.gov/bioproject/PRJNA1145605.

## Footnotes

^1^ https://qiime2.org/
