# Supplementary material for: Niche-specification of aerobic 2,4-dichlorophenoxyacetic acid biodegradation by tfd-carrying bacteria in the rice paddy ecosystem
Source: Front Microbiol. 2024 Aug 23;15:1425193. doi: 10.3389/fmicb.2024.1425193 (PMC11377324; doi:10.3389/fmicb.2024.1425193)
Supplement: Supplementary file 1 [file Data_Sheet_1.pdf]

Supplementary Material

1 Supplementary Data

Data (Tables S2, S3A, S3B, S3C, S4A, and S4B) are presented in Excel files.

2 Supplementary Figures and Tables

2.1 Supplementary Figures

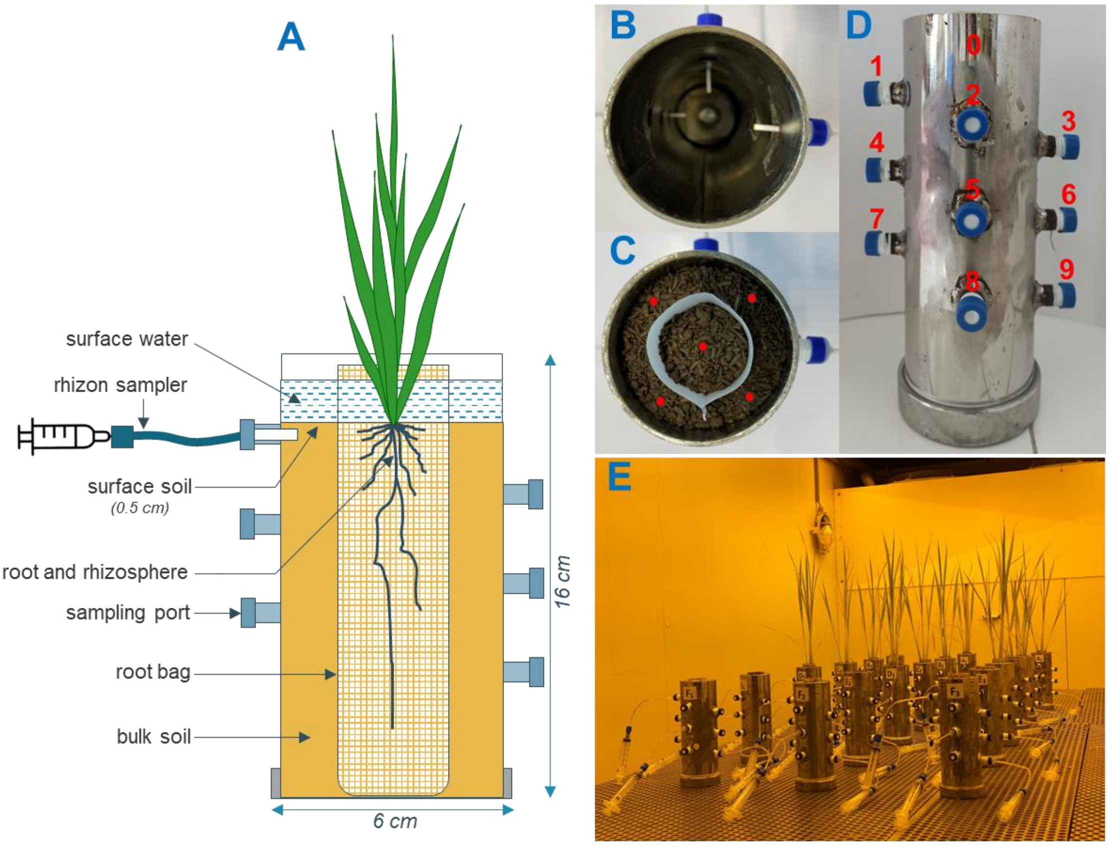

Supplementary figure S1: Schematic presentation of the used rice paddy soil microcosm setup. **(A)** the overall structure of the rice paddy microcosm containing the rice plant indicating relevant attributes and the different compartments. **(B)** Top view of the rice paddy soil microcosm without soil. **(C)** Top view of the rice paddy soil microcosm with soil and root showing the positions (red spots) used for injecting the 2,4-D solution. **(D)** Positions of the water sampling ports along the length of the microcosm. The ports were numbered from top to bottom. **(E)** Rice paddy microcosm setup incubated in the plant growth chamber.

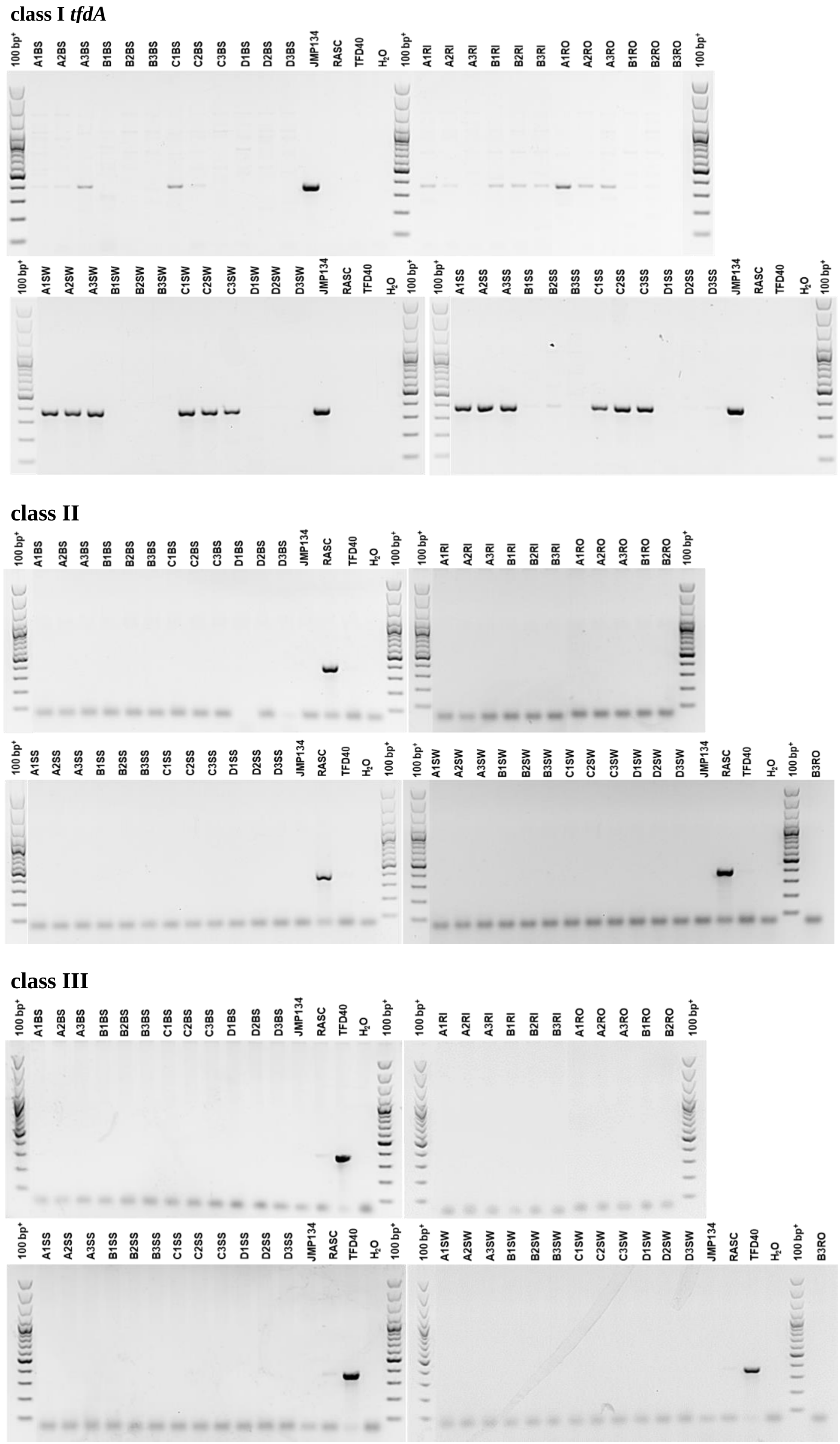

Supplementary figure S2: End-point PCR detection of class I, class II and class III *tfdA* variants in total community DNA derived from the different treatments in the microcosm

experiment. Lanes: Treatments are indicated as follows: A, “Soil + rice plant + 2,4-D”; B, “Soil + rice plant”; C, “Soil + 2,4-D”; D, “Soil”. The numbers 1, 2 and 3 indicate the respective replicates. The niches/compartments are indicated as follows: SW, surface water; SS, surface soil; RI, rhizosphere; RO, root surface; BS, bulk soil. JMP134: DNA extracted from *Cupriavidus necator* JMP134 (carries class I *tfdA*), RASC: DNA extracted from *Burkholderia* sp. RASC (carries class II *tfdA*), TFD40: DNA extracted from *Cupriavidus* sp. TFD40 (carries class III *tfdA*).

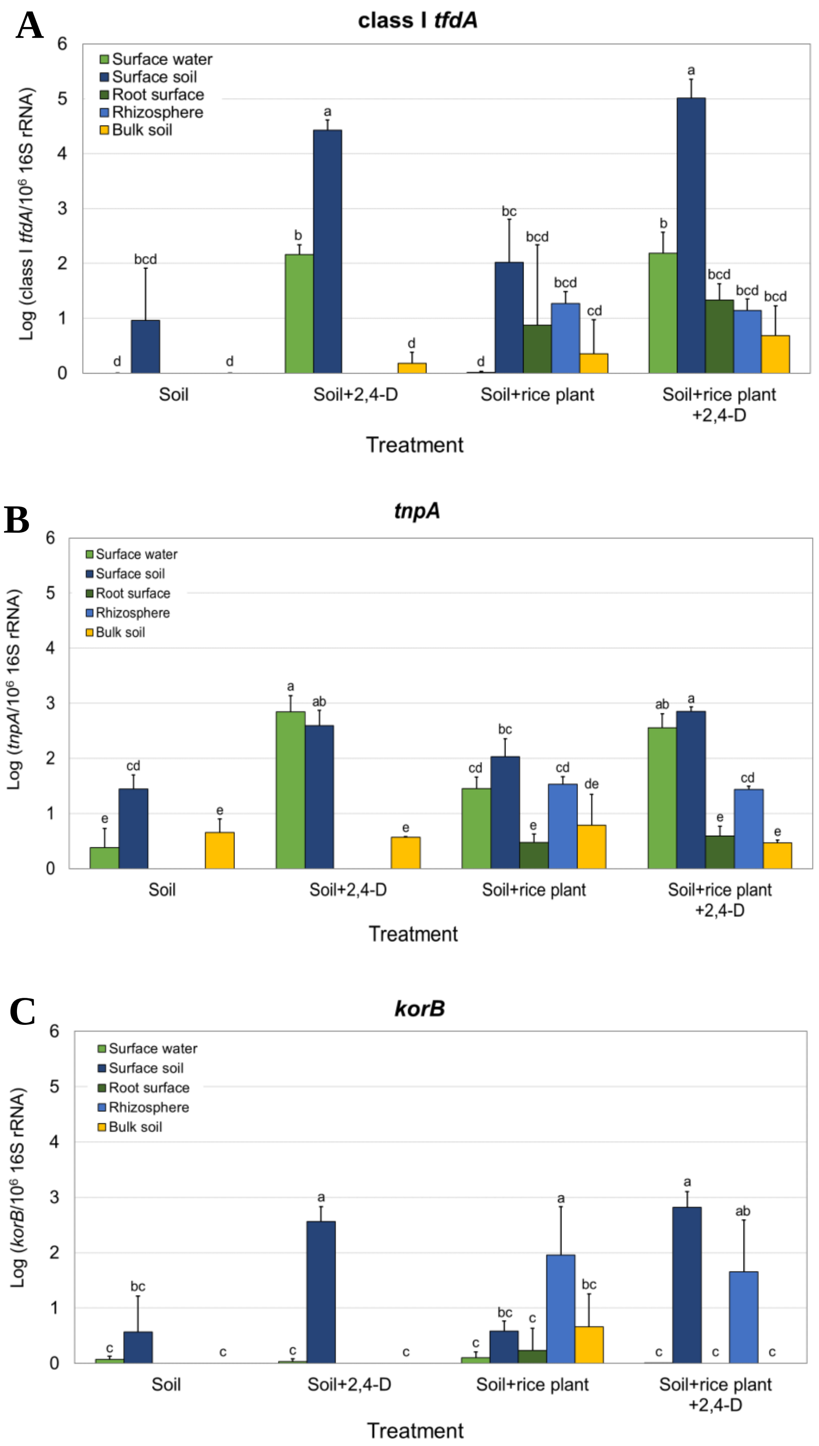

Supplementary figure S3: Relative abundances of class I *tfdA* **(A)**, IS*1071* (*tnpA*) **(B)** and IncP-1 plasmids (*korB*) **(C)** in the different niches for each treatment in the microcosm experiment as determined by targeted qPCR. Relative abundances are expressed as the log of the ratios of the number of target gene copies over the number of 10^6 16S rRNA gene copies. Values indicated are the average of three replicates with standard deviation. Different letters in each treatment indicate values that are significantly different over all treatments (ANOVA, $P<0.05$).

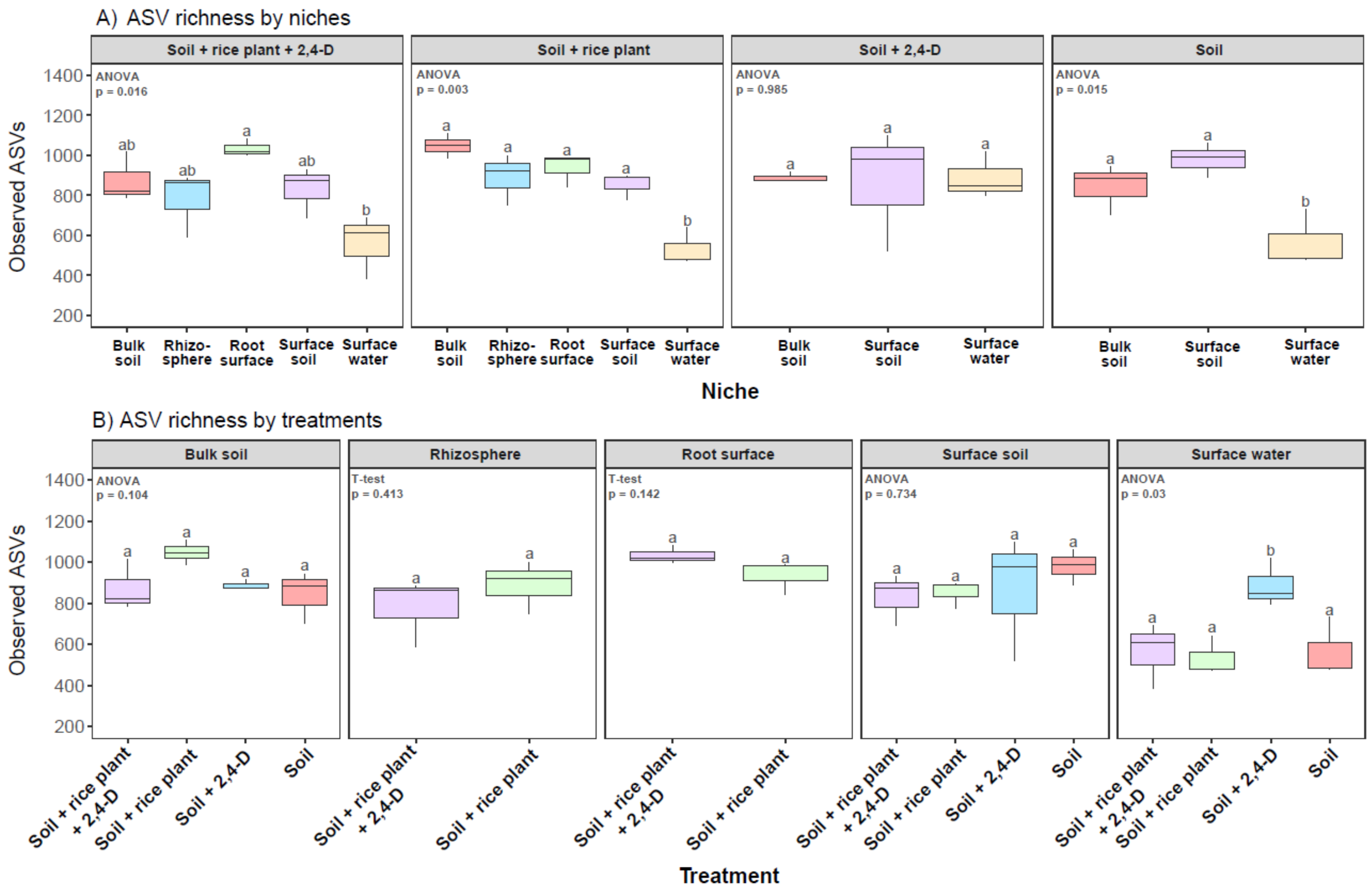

Supplementary figure S4: Box plots of α-diversity (ASV richness) in the different niches for each treatment **(A)** and in the different treatments for each niche **(B)** in the microcosm experiment as determined by bacterial 16S rRNA gene amplicon sequencing. Box plot represents: whiskers = minimum and maximum values, the box = the range between 1st (lower) and 3rd (upper) quartiles, horizontal bold line = median. Different letters indicate values that are significantly different between the niches in one treatment in **(A)** and between the treatments in one niche in **(B)** (ANOVA/T-test, P<0.05).

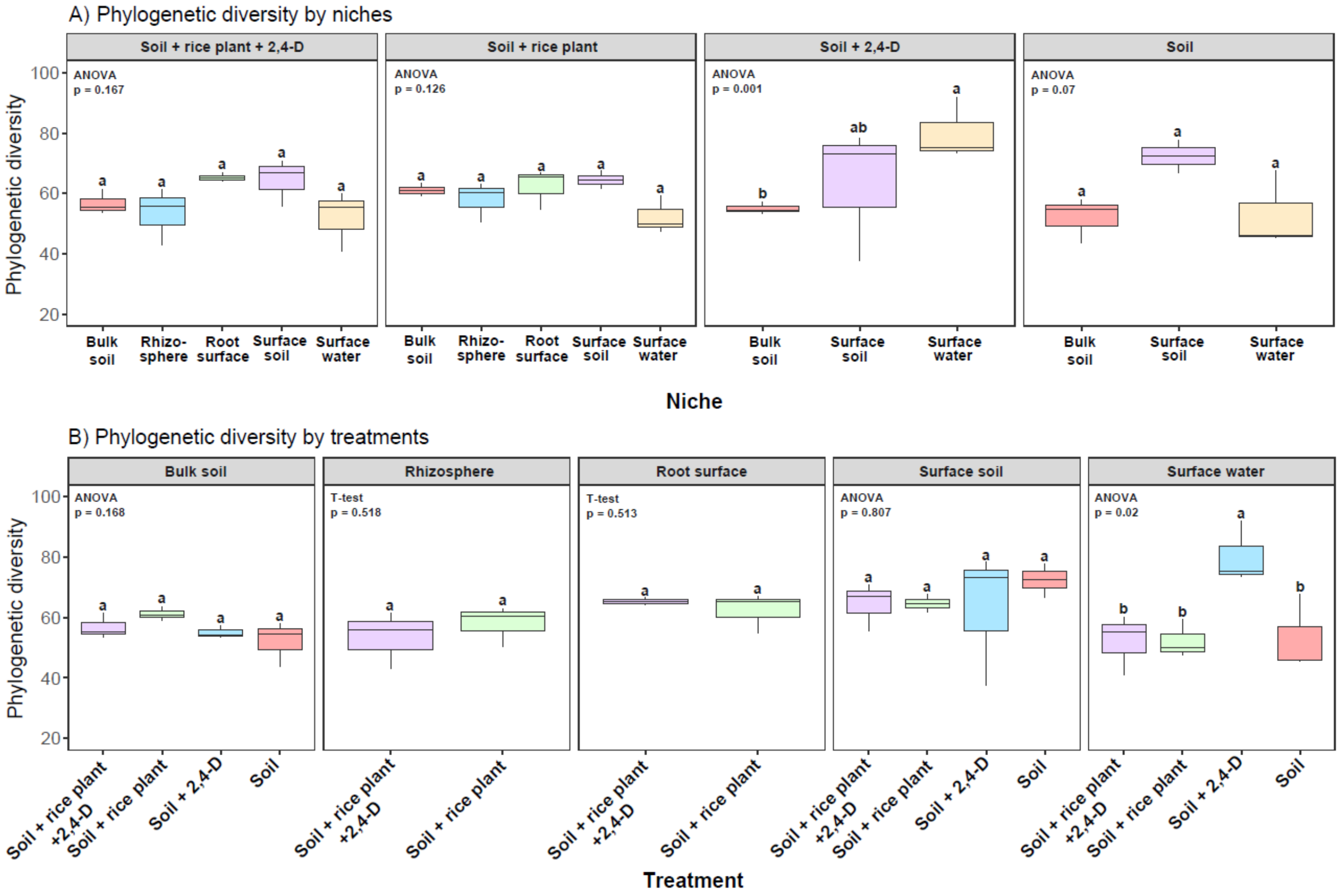

Supplementary figure S5: Box plots of α-diversity (phylogenetic diversity) in the different niches for each treatment **(A)** and in the different treatments for each niche **(B)** in the microcosm experiment as determined by bacterial 16S rRNA gene amplicon sequencing. Box plot represents: whiskers = minimum and maximum values, the box = the range between 1st (lower) and 3rd (upper) quartiles, horizontal bold line = median. Different letters indicate values that are significantly different between the niches in one treatment in **(A)** and between the treatments in one niche in **(B)** (ANOVA/T-test, *P*<0.05).

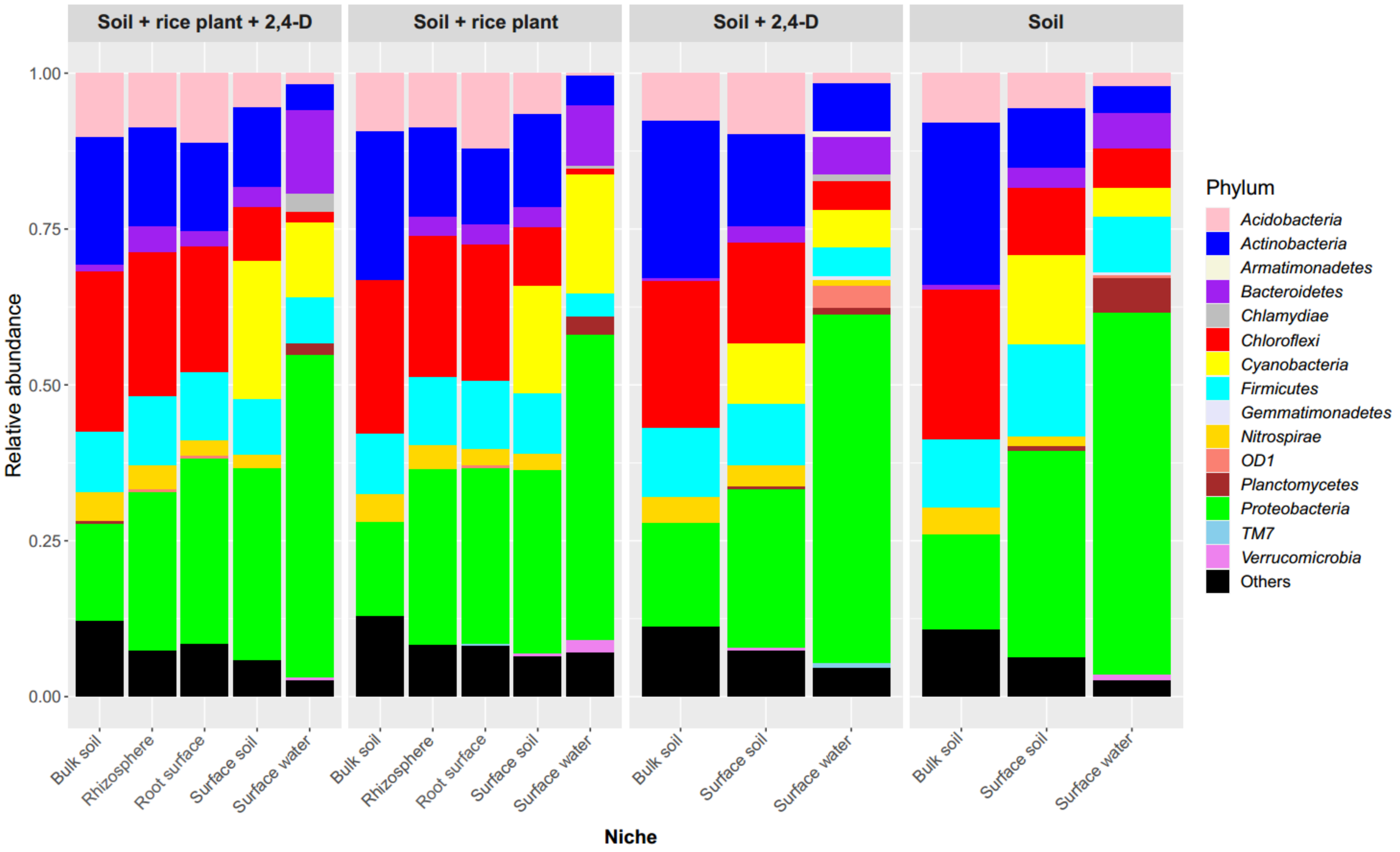

Supplementary figure S6: Bar plot showing the relative abundances of bacterial phyla in bulk soil, rhizosphere, root surface, surface soil and surface water across the treatments in the microcosm experiment as determined by bacterial 16S rRNA gene amplicon sequencing.

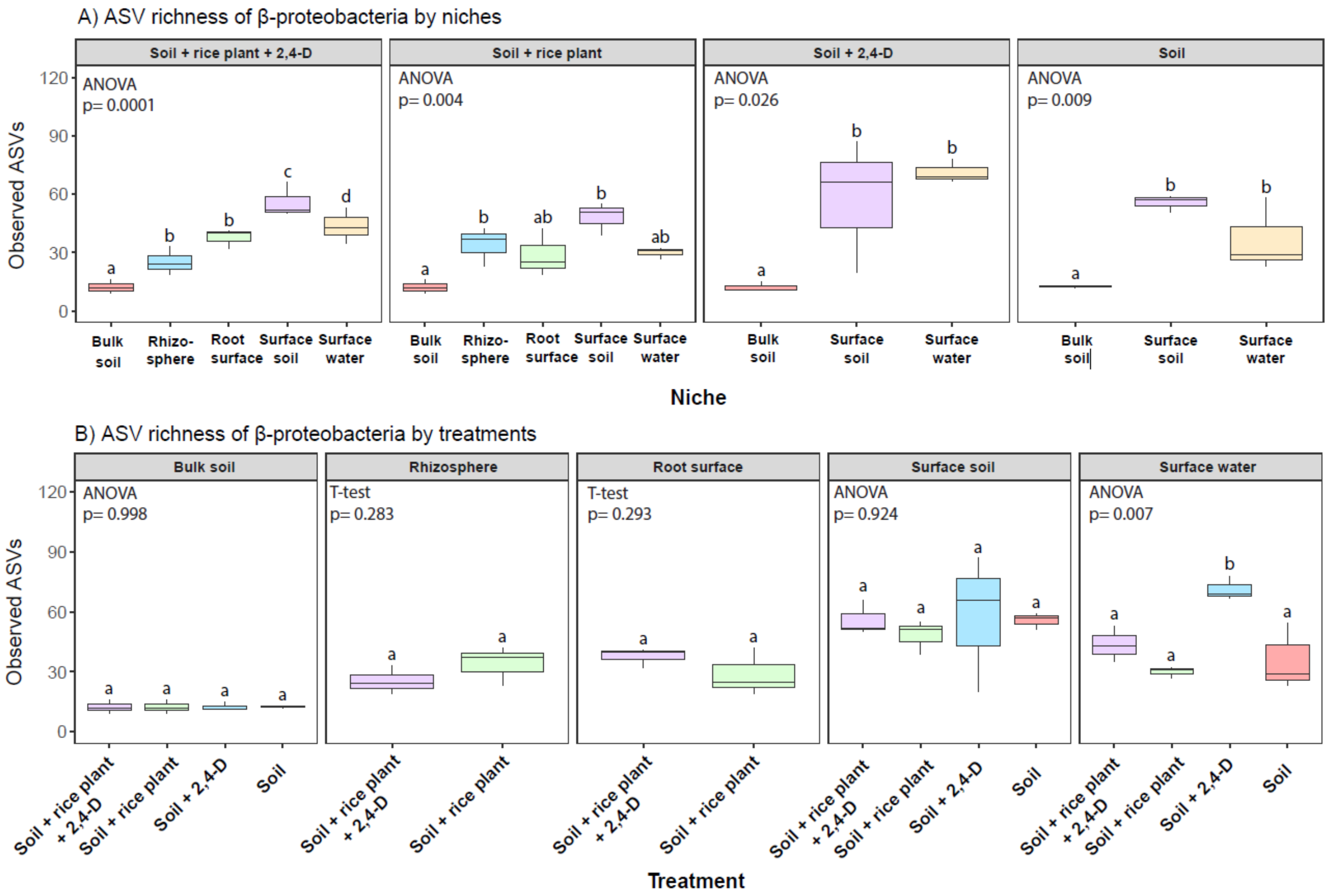

Supplementary figure S7: Box plots of α-diversity (ASV richness) of β-proteobacteria in the different niches for each treatment **(A)** and in the different treatments for each niche **(B)** as determined by bacterial 16S rRNA gene amplicon sequencing. Box plot represents: whiskers = minimum and maximum values, the box = the range between 1st (lower) and 3rd (upper) quartiles, horizontal bold line = median. Different letters indicate values that are significantly different between the niches in one treatment in **(A)** and between the treatments in one niche in **(B)** (ANOVA/T-test, $P<0.05$).

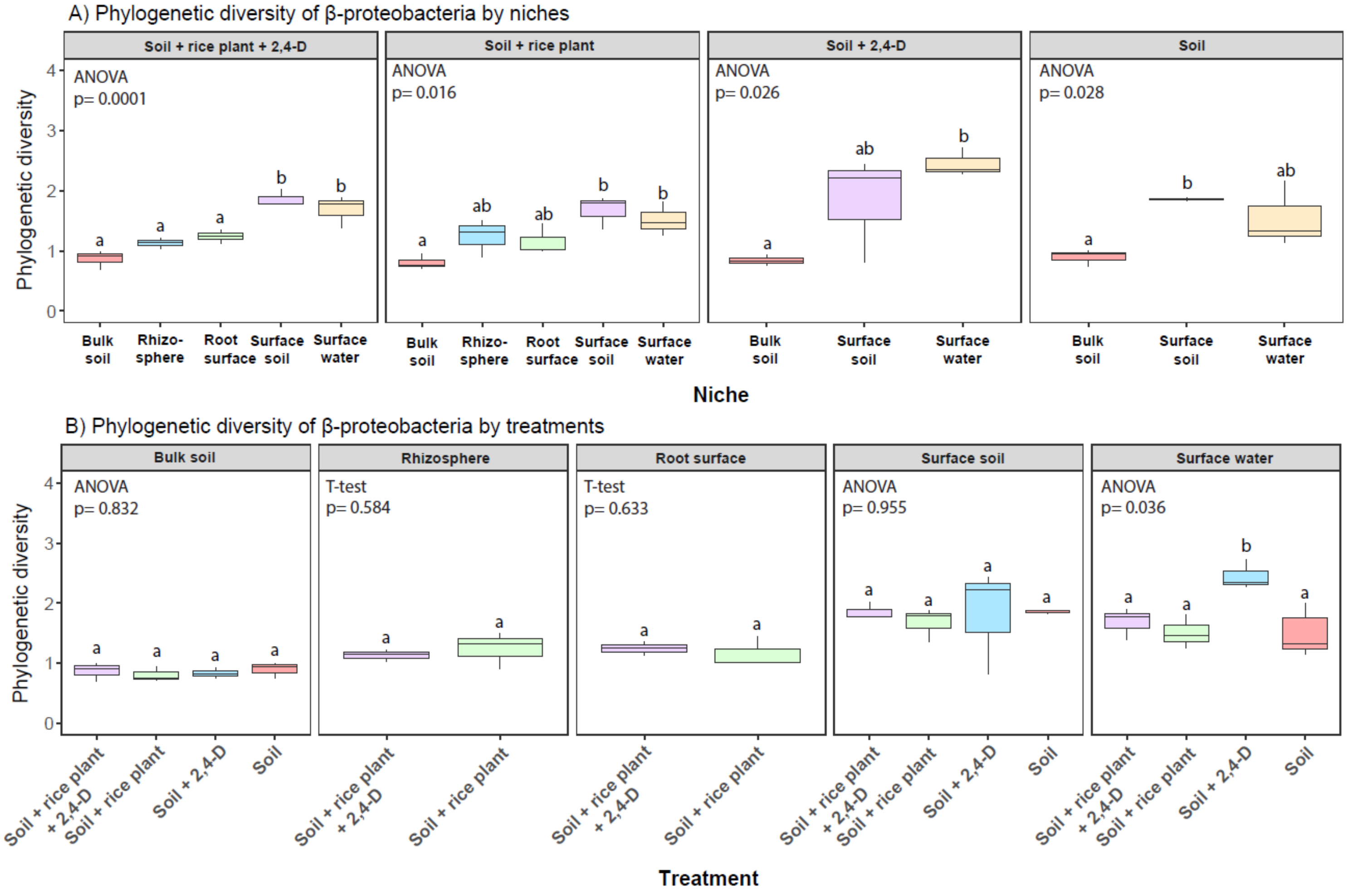

Supplementary Figure S8: Box plots of α-diversity (phylogenetic diversity) of β-proteobacteria in the different niches for each treatment **(A)** and in the different treatments for each niche **(B)** as determined by bacterial 16S rRNA gene amplicon sequencing. Box plot represents: whiskers = minimum and maximum values, the box = the range between 1st (lower) and 3rd (upper) quartiles, horizontal bold line = median. Different letters indicate values that are significantly different between the niches in one treatment in **(A)** and between the treatments in one niche in **(B)** (ANOVA/T-test, *P*<0.05).

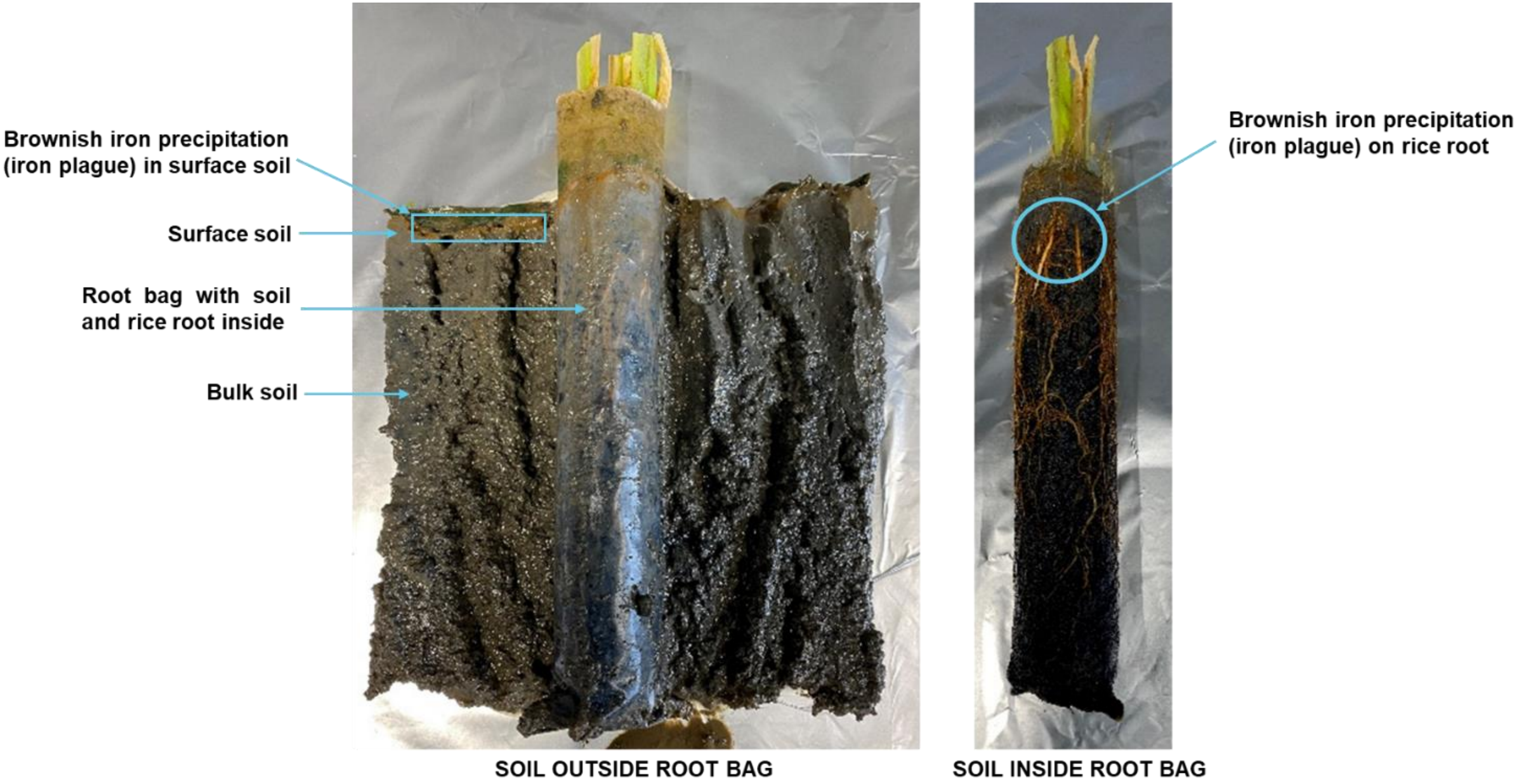

Supplementary Figure S9: The appearance of brownish iron plaque observed in surface soil and at the level of the rice root.

2.2 Supplementary Tables

Supplementary Table S1: Overview of the oligonucleotide sequences of the primers and probes used in this study for performing end-point PCR/qPCR targeting the bacterial 16S rRNA gene, *tfdA* (class I, II and III), *tnpA* (as marker of IS*1071*), *korB* (as marker of IncP-1 plasmids) and used PCR conditions.

Primers/probes (5'-3')	Step 1	Step 2	Step 3	Step 4	Step 5	Cycles	References
End-point PCR							
**Class I *tfdA***							
tfdA_class_I_F: GTGAGCGTCGTCGCAAAT<br>tfdA_class_I_R: GCATCGTCCAGGGTGGTC	95ºC<br>10 min	95ºC<br>45 sec	58ºC<br>45 sec	72ºC<br>90 sec	72ºC<br>7 min	25x<br>(steps 2-4)	(Baelum & Jacobsen, 2009)
**Class II *tfdA***							
tfdA_class_II_F: TGAGCATCAATTCCGAATACC<br>tfdA_class_II_R: AAGACTGACCCCGTGGACT	95ºC<br>10 min	95ºC<br>45 sec	55ºC<br>45 sec	72ºC<br>90 sec	72ºC<br>7 min	25x<br>(steps 2-4)	
**Class III *tfdA***							
tfdA_class_III_F: TGAGCATCACTTCCGAATACC<br>tfdA_class_III_R: ACAGCGTCGTCCAACGTC	95ºC<br>10 min	95ºC<br>45 sec	58ºC<br>45 sec	72ºC<br>90 sec	72ºC<br>7 min	25x<br>(steps 2-4)	
qPCR							
**16S rRNA**							
341F: CCTAYGGGRBGCASCAG<br>534R: ATTACCGCGGCTGCTGGC	95ºC<br>15 min	95ºC<br>20 sec	60ºC<br>20 sec	72ºC<br>20 sec	-	40x<br>(steps 2-4)	(Haest *et al.*, 2011)
***tnpA* gene (IS*1071*)**							
IS1071_F2: CATGATGCCGGCRTAGACCA<br>IS1071_R2: GAAGTGATYCTGGCCGTTGAC	95ºC<br>15 min	95ºC<br>10 sec	64ºC<br>10 sec	72ºC<br>15 sec	-	40x<br>(steps 2-4)	This study
**Class I *tfdA***							
tfdA_81bp_F: GAGCACTACGCRCTGAAYTCCCG<br>tfdA_81bp_R: SACCGGMGGCATSGCATT<br>tfdA_class1: 6-FAM-TTGCGCTTCCGAATAGTCGGTGTC-BHQ1	95ºC<br>15 min	95ºC<br>30 sec	62ºC<br>90 sec	72ºC<br>7 min	-	50x<br>(steps 2-3)	(Baelum & Jacobsen, 2009)
***korB* (IncP-1)**							
korB_F: TCATCGACAACGACTACAACG<br>korB_Fz: TCGTGGATAACGACTACAACG<br>korB_R: TTCTTCTTGCCCTTCGCCAG<br>korB_Rge: TTYTTCYTGCCCTTGGCCAG<br>korB_Rd: TTCTTGACTCCCTTCGCCAG<br>korB P: 6-FAM-TCAGYTCRTTGCGYTGCAGGTTCTCVAT-TAM<br>korB Pgz: 6-FAM-TSAGGTCGTTGCGTTGCAGGTTYTCAAT-TAM	95ºC<br>10 min	95ºC<br>15 sec	54ºC<br>15 sec	60ºC<br>60 sec	-	40x<br>(2-4)	(Jechalke *et al.*, 2013)

3 Supplementary Methods

3.1 Bacterial community analysis

16S rRNA gene amplicon sequence reads from DNA extracts from the microcosm samples were obtained using DNA Nanoball Sequencing (BGI Genomics, China). Reads were provided by BGI for each sample with the adaptor and primer sequences already removed. The sequences were dereplicated, denoised, and paired-end merged using the DADA2 pipeline v.1.18.0 with Phred score ≥ 25 (Callahan *et al.*, 2016). Chimeric sequences and singletons with a truncation length of zero were removed using the same DADA2 pipeline and the remaining sequences clustered into Amplicon Sequence Variants (ASVs), providing feature tables and representative sequences as outputs. The representative sequence sets were each aligned by MAFFT v.7.475 (Katoh & Standley, 2013) from which a rooted and unrooted phylogenetic tree was created by FastTree v.2.1.11 (Price (Price *et al.*, 2010) *et al.*, 2010). Taxonomic classification of the representative sequence for each ASV was done using the QIIME's version of Ribosomal Database Project's classifier(McDonald *et al.*, 2023) against the pre-trained Greengenes 16S rRNA (13_8 release) at 99% nucleotide sequence similarity.

References

Baelum J & Jacobsen CS (2009) TaqMan Probe-Based Real-Time PCR Assay for Detection and Discrimination of Class I, II, and III tfdA Genes in Soils Treated with Phenoxy Acid Herbicides. *Appl Environ Microb* **75**: 2969-2972.

Callahan BJ, Sankaran K, Fukuyama JA, McMurdie PJ & Holmes SP (2016) Bioconductor Workflow for Microbiome Data Analysis: from raw reads to community analyses. *F1000Res* **5**: 1492.

Haest PJ, Philips J, Springael D & Smolders E (2011) The reactive transport of trichloroethene is influenced by residence time and microbial numbers. *Journal of Contaminant Hydrology* **119**: 89-98.

Jechalke S, Dealtry S, Smalla K & Heuer H (2013) Quantification of IncP-1 plasmid prevalence in environmental samples. *Appl Environ Microbiol* **79**: 1410-1413.

Katoh K & Standley DM (2013) MAFFT multiple sequence alignment software version 7: improvements in performance and usability. *Mol Biol Evol* **30**: 772-780.

McDonald D, Jiang Y, Balaban M, *et al.* (2023) Greengenes2 unifies microbial data in a single reference tree. *Nature Biotechnology*.

Price MN, Dehal PS & Arkin AP (2010) FastTree 2--approximately maximum-likelihood trees for large alignments. *PLoS One* **5**: e9490.
